# Discovery of Influenza A Virus Sequence Pairs and Their Combinations for Simultaneous Heterosubtypic Targeting that Hedge against Antiviral Resistance

**DOI:** 10.1371/journal.pcbi.1004663

**Published:** 2016-01-15

**Authors:** Keng Boon Wee, Raphael Tze Chuen Lee, Jing Lin, Zacharias Aloysius Dwi Pramono, Sebastian Maurer-Stroh

**Affiliations:** 1 Fluid Dynamics Department, Institute of High Performance Computing (IHPC), A*STAR (Agency for Science, Technology and Research), Singapore, Singapore; 2 Biomolecular Function Discovery Division, Bioinformatics Institute (BII), A*STAR (Agency for Science, Technology and Research), Singapore, Singapore; 3 Department of Research, National Skin Centre, Singapore, Singapore; 4 National Public Health Laboratory, Ministry of Health, Singapore, Singapore; 5 School of Biological Sciences, Nanyang Technological University, Singapore, Singapore; University of California San Diego, UNITED STATES

## Abstract

The multiple circulating human influenza A virus subtypes coupled with the perpetual genomic mutations and segment reassortment events challenge the development of effective therapeutics. The capacity to drug most RNAs motivates the investigation on viral RNA targets. 123,060 segment sequences from 35,938 strains of the most prevalent subtypes also infecting humans–H1N1, 2009 pandemic H1N1, H3N2, H5N1 and H7N9, were used to identify 1,183 conserved RNA target sequences (≥15-mer) in the internal segments. 100% theoretical coverage in simultaneous heterosubtypic targeting is achieved by pairing specific sequences from the same segment (“*Duals*”) or from two segments (“*Doubles*”); 1,662 *Duals* and 28,463 *Doubles* identified. By combining specific *Duals* and/or *Doubles* to form a target graph wherein an edge connecting two vertices (target sequences) represents a *Dual* or *Double*, it is possible to hedge against antiviral resistance besides maintaining 100% heterosubtypic coverage. To evaluate the hedging potential, we define the hedge-factor as the minimum number of resistant target sequences that will render the graph to become resistant i.e. eliminate all the edges therein; a target sequence or a graph is considered resistant when it cannot achieve 100% heterosubtypic coverage. In an *n*-vertices graph (*n ≥ 3*), the hedge-factor is maximal (= *n– 1*) when it is a complete graph i.e. every distinct pair in a graph is either a *Dual* or *Double*. Computational analyses uncover an extensive number of complete graphs of different sizes. Monte Carlo simulations show that the mutation counts and time elapsed for a target graph to become resistant increase with the hedge-factor. Incidentally, target sequences which were reported to reduce virus titre in experiments are included in our target graphs. The identity of target sequence pairs for heterosubtypic targeting and their combinations for hedging antiviral resistance are useful toolkits to construct target graphs for different therapeutic objectives.

## Author Summary

An average of three influenza pandemics occurred in each century over the last 300 years. As occurrence of the next influenza pandemic is definite, developing new antivirals is imperative since resistance to the remaining class of antivirals has been reported occasionally, and vaccines are ineffective in the initial wave of a pandemic. The typical evolutionary traits of viruses, which manifest as multiple virus subtypes in circulation and perpetual viral genomic mutations, require the development of subtype-specific antivirals that ultimately acquire resistance. Being a rapidly evolving and highly contagious virus that manifest the most subtypes, this is particularly acute for influenza A. Our approach to overcome these challenges is to identify and characterize influenza A virus sequences for RNA targeting that can theoretically address all strains from the most prevalent human-infecting subtypes (i.e. simultaneous multi-subtype targeting) that can hedge against antiviral resistance. We uncover an extensive list of target sequence pairs and their specific combinations for which they can be selected for novel therapeutics development that will be effective on multiple circulating seasonal strains and future pandemic strains. As our approach is applicable to other viruses, the methods are general for use in the selection of antiviral therapeutic targets.

## Introduction

An average of three influenza pandemics occurred in each century over the last 300 years [1]. The time interval between consecutive pandemics and their respective mortality are however irregular; while the 1918 H1N1 Spanish flu was estimated to kill 50 million people, the 2009 H1N1 Swine flu pandemic was probably responsible for 100,000 to 200,000 deaths [2]. As substitution of a few specific amino acids can be sufficient to alter host tropism [3,4], it is relatively easy for a novel influenza A viral subtype previously circulating in animals against which the general human population lacks antibody-mediated immunity to cause future pandemics.

Vaccines and anti-viral drugs respectively are the main biologics and pharmaceuticals tools to reduce the morbidity and mortality of a pandemic. Depending on the circulating viral strains and the recipients’ age, vaccine effectiveness can be lower than 40% [5]. Moreover, vaccines are unlikely to be available in the initial wave of a pandemic as current vaccine approaches are lineage and subtype-specific and such vaccines are typically developed after the new antigenically distinct pandemic virus has emerged. Anti-viral drugs are classified by their target viral proteins, typically–M2 ion-channel inhibitors (amantadine and rimantadine) and neuraminidase inhibitors (e.g. oseltamivir and zanamivir) [6]. The former are now ineffective against current circulating H3N2 and H1N1 (2009) subtypes [7–9]. Neuraminidase inhibitors are the sole antiviral option in a pandemic while incidences of resistance have been reported occasionally [10–15]. When coupled with so-called permissive mutations, the classical Tamiflu-resistance mutation H274Y (H275Y) can become more prevalent and in the case of the previous seasonally circulating H1N1 virus, became fixed in circulating viruses rapidly in 2008 [16]. Developing new antivirals to anticipate resistance in seasonal as well as potential future pandemic viruses is thus imperative. Unfortunately, the multiple circulating influenza A virus subtypes coupled with the perpetual genomic mutations and segment reassortment events challenge the development of effective therapeutics against multiple circulating and future strains.

An arsenal of protein inhibitors that each binds to distinct sites of all expressed viral proteins is one strategy for heterosubtypic targeting and to hedge against inevitable antiviral resistance. While chemical protein inhibitors constitute most pharmaceuticals, their targets are limited [17–18]. Alternatively, prior studies have demonstrated viral RNA targeting via siRNA or antisense oligonucleotides (AONs) as viable antiviral strategies [19–42]. They can potentially target any sequences within a viral RNA segment leading to RNA degradation that is elicited by either RNAi or RNase-H, or inhibition of RNA splicing or translation by steric hindrance effects [43]. Of particular clinical relevance is the demonstration that intranasal AON inhalation is an efficient delivery vehicle to the respiratory tract and lungs in animal studies [21,24,27–28,35]. Additionally, rational computational methods [44] to identify optimum RNA target sites can facilitate rapid development of an AON library for hedging against antiviral resistance and for targeting novel viral strains in a pandemic.

To examine heterosubtypic RNA targeting, we identify and characterize conserved RNA target sequences in the eight influenza A virus segments from subtypes infecting humans and animals. Analyses on 168,986 segment sequences derived from 51,661 human and animal strains reveal thousands of specific pairs of target sequences that can address all prevalent circulating human strains simultaneously. Novel strategies for target sequence selection to hedge against antiviral resistance illuminate countless sets of target sequence combinations with distinct hedging capacities. Together, the target sequences and their specific combinations discovered in this pan-virus subtype study is a useful resource for the development of effective RNA therapeutics, which targets viral RNA, mRNA or cRNA, against multiple circulating and future strains.

## Results

### Identification of conserved RNA target sequences

Five subtypes representing the most prevalent human infecting Influenza A viruses in the past four decades were studied–H1N1 (before 2009; hitherto refer to as H1N1), 2009 pandemic H1N1pdm09 (hitherto refer to as PD09), H3N2, H5N1 and H7N9. Although both H5N1 and H7N9 subtypes are primarily avian influenza viruses, they have periodically caused human infections with occasional reports of human adaptive mutations and therefore pose a significant risk of pandemic potential. For each subtype, all available sequences of each of the eight viral segments were downloaded from curated databases (refer to S1 Text). 123,060 segment sequences from 35,938 strains of which 70,723 are unique were analysed (S1 Table breakdowns the sequence counts by subtype and segment).

**Fig 1:**
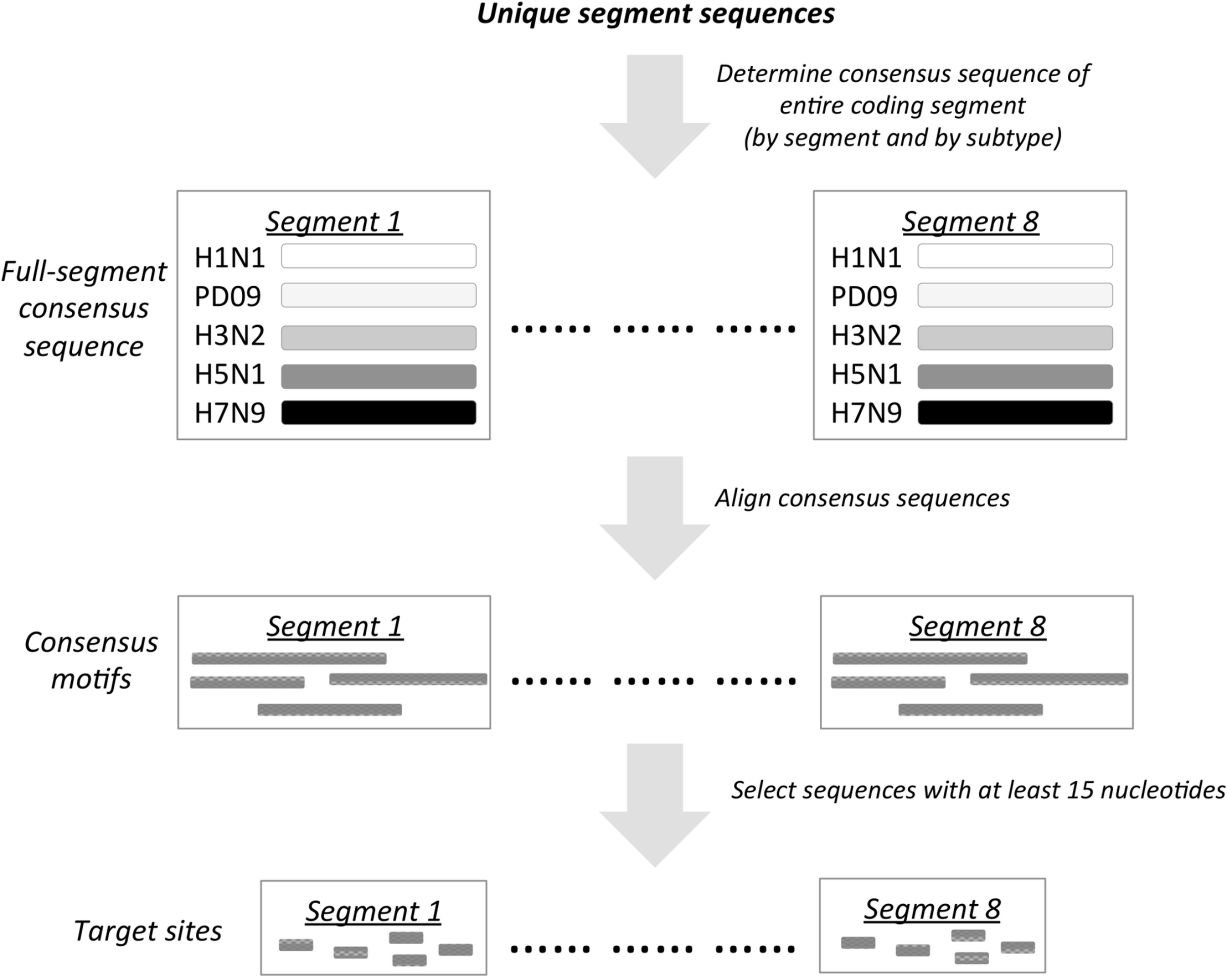
Schematic to obtain 5-S target sequences.

Two sets of RNA target sequences were obtained. Sequences in the “5-S” set were selected to optimally target the five subtypes simultaneously whereas sequences in the “3-S” set were selected to target H1N1, PD09 and H3N2 simultaneously. The 5-S set was obtained as depicted in Fig 1. First, the consensus sequence of the entire coding segment was determined for every segment of each subtype from the respective unique sequences. Next, for each segment, sequence alignment was performed on all the respective consensus sequences from the five subtypes simultaneously. Consensus motifs defined as sections of the consensus sequence with perfect alignments were identified. Finally, target sequences of at least 15 nucleotides were selected from the respective collection of consensus motifs in each segment; this minimum target length is chosen for RNA binding specificity and thermodynamic stability. The 3-S set was obtained similarly by omitting H5N1 and H7N9 segment sequences. Section A in Table 1 summarizes both the 5-S and 3-S sets; note that 5-S is a subset of 3-S and the much smaller number of conserved target sequences in 5-S illustrates, not surprisingly, that target sequence conservation strongly depends on the number and selection of strains. Both segments 4 and 6, which code for the more variable hemagglutinin and neuraminidase surface proteins respectively, cannot be targeted as they do not share any 15-mer sequences between the subtypes consensus. When the target sequences counts were normalized with the respective segment coding lengths, conserved target sequences appear enriched in segment 7 (coding for M1 and M2 proteins in alternative frames) in 5-S and 3-S, and in segment 1 (coding for PB2 protein) of 3-S.

**Table 1:** Single target sequences and *Duals* in 5-S and 3-S sets.

**A**	***5-S***			***3-S***		
**Segment**	**Counts**	**Counts (Normalized)**	**Maximum Length**	**Counts**	**Counts (Normalized)**	**Maximum Length**
**1**	156	0.068	29	2,722	1.194	86
**2**	243	0.107	34	279	0.123	34
**3**	49	0.023	20	493	0.229	35
**4**	0	–	–	0	–	–
**5**	107	0.071	26	281	0.188	26
**6**	0	–	–	0	–	–
**7**	594	0.605	44	1,696	1.727	68
**8**	34	0.041	21	52	0.063	21
**Total**	**1,183**			**5,523**		
**B**	***5-S***			***3-S***		
**Segment**	***Duals* Counts**	***Singles* Counts**	***Singles (*%)**	***Duals* Counts**	***Singles* Counts**	***Singles (*%)**
**1**	943	156	100%	15,363	1340	49.23%
**2**	36	12	4.9%	42	13	4.66%
**3**	96	28	57%	6,971	394	79.92%
**5**	–	–	–	4,167	140	49.82%
**7**	587	81	14%	2,578	296	17.45%
**8**	–	–	–	3	4	7.69%
**Total**	**1,662**	**277**	**23%**	**29,124**	**2,187**	**40%**

**(A) Single target sequences**. Target sequence counts were also normalized with the respective segment coding lengths (columns 3 and 6). **(B) Effective *Duals***. The numbers of effective *Duals* (that can cover all unique sequences of respective segments) are listed as “*Duals* Counts”. The single target sequences that constitute these *Duals* are also given as “*Singles* Counts” and “*Singles* %” (percent of total single target sequences tabulated in Section A in Table 1).

To evaluate coverage of intra-subtype variation for every conserved target sequence in a segment, it was matched against every unique sequence of the respective segment from five subtypes in 5-S or from three subtypes (H1N1, PD09 and H3N2) in 3-S. No target sequence was found in all relevant unique segment sequences although there are always some in each target segment that are found in more than 95% of the respective unique sequences (S1 and S2 Figs). The coverage against human corresponding animal subtypes (aH1N1 aH3N2 aH5N1 and aH7N9) and three groups of collective subtypes labelled as “*H00N00*”, “*zoonotic*” and “*exotic*” (Materials and Methods) were also determined. The *H00N00* group consists of eight subtypes that have infected humans but are not or no longer in large-scale human circulation whereas the *zoonotic* and *exotic* groups respectively consist of 78 and 19 animal subtypes with zoonotic potential. 45,926 sequences from the six internal segments of which 32,961 are unique, were consolidated from 15,728 strains and analysed (S2 Table). Notably, there are human target sequences that are found in more than 90% of the unique sequences in each target segment of each animal subtype and in each group of subtypes (S3 and S4 Figs present the coverage of every target sequence against each human and corresponding animal subtypes, and against each group of subtypes). Hence, both 5-S and 3-S sets are relatively conserved in a total of 109 human and animal subtypes; for more coverage analyses, see S5 and S6 Figs. Coverage against Influenza B virus was 0%. In order to achieve 100% coverage in human subtypes, we next considered target sequence pairs.

### Target sequence pairs within a segment (“*Duals*”)

Target sequences within a segment were paired (each pair is termed a *Dual*). In the 5-S set, effective *Duals* in four segments (1, 2, 3 and 7) and in the 3-S set, effective *Duals* in six internal segments can cover all unique sequences of respective target segments (Section B in Table 1). That is, one or both of the target sequences constituting an effective *Dual* are found in all unique segment sequences. A significant fraction of single target sequences in segments 1, 3 and 5 forms effective *Duals* (Section B in Table 1). The distribution of the target sequence positions from the effective *Duals* in each segment are depicted in S7 Fig.

**Fig 2:**
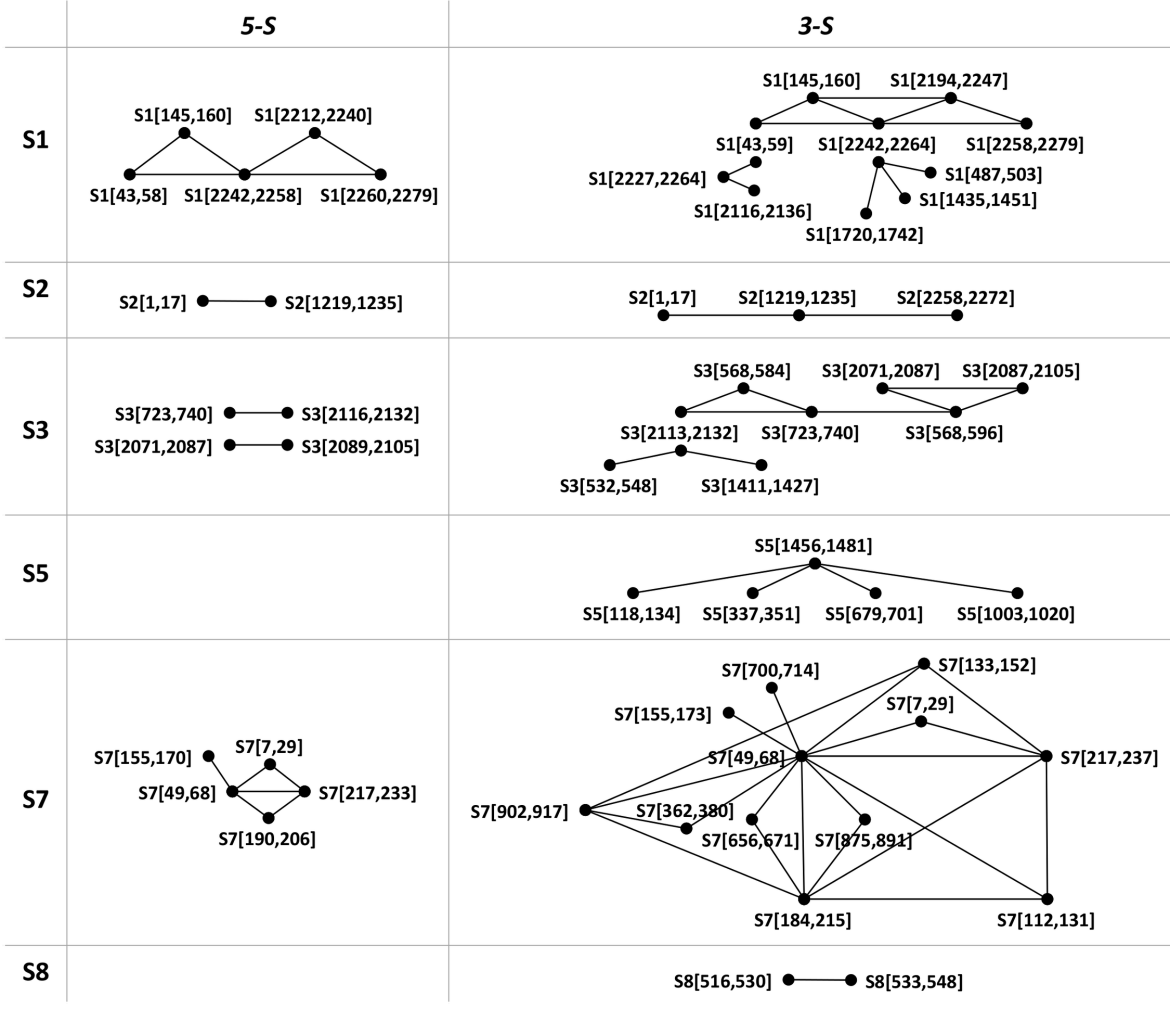
Clusters of effective *Duals*. Each vertex denotes a cluster of overlapping single target sequences whose first and last positions were given within the enclosing square brackets. An edge connecting two vertices signifies that target sequences between the two clusters form one or more effective *Duals*. Note: overlapping clusters in S1 (3-S) are not merged as a cluster because they can form effective *Duals*.

Next, overlapping single target sequences in a segment (tallied in Section B in Table 1) were grouped as clusters. Two clusters are paired when target sequences between the two clusters form one or more effective *Duals*. The cluster pairings in both 5-S and 3-S are depicted as graphs in which a pairing is denoted by an undirected edge connecting two clusters depicted as vertices (Fig 2). The utilization of cycle graphs in Fig 2 (i.e. vertices connected in a closed chain) in selecting effective *Duals* for hedging against antiviral resistance will be discussed.

### Target sequence pairs between two segments (“*Doubles*”)

Alternatively, target sequences from two segments were paired (each pair is termed a *Double*). A *Double* can target a virus strain when one or both of its target sequences is found in either one or both of the strain’s respective segment sequences. The coverage of a *Double* is defined as the fraction of total virus strains in five subtypes in 5-S or in three subtypes in 3-S that it can target. The coverage of every *Double* was determined for every segment pairing (each with at least 10,000 strains, S3 Table). As summarized in Table 2, for each of the 15 target segment pairings, there are effective *Doubles* with 100% strain coverage. In contrast to *Duals* (Section B in Table 1), most of the single target sequences are included in effective *Doubles* (Table 3). The collective distribution of the target sequence positions from effective *Doubles* in each segment are given in S8 Fig.

**Table 2:** *Doubles* in 5-S and 3-S sets.

	*5-S*					*3-S*				
Segment	2	3	5	7	8	2	3	5	7	8
**1**	510	645	6,821	5,853	117	7,598	102,840	36,287	63,335	4,831
**2**	–	414	2,280	2,634	72	–	4,107	4,482	3,828	162
**3**	–	–	3,687	879	108	–	–	28,971	11,710	2,233
**5**	–	–	–	2,946	468	–	–	–	6,807	1,404
**7**	–	–	–	–	1,029	–	–	–	–	1,756
**Total**	**28,463**					**280,351**				

**T**he number of effective *Doubles* (with 100% virus strain coverage) in each of the total of 15 segment pairings.

**Table 3:** Single target sequences constituting the *effective* Doubles in 5-S and 3-S sets.

	*5-S*		*3-S*	
Segment	*Singles* Counts	*Singles (*%)	*Singles* Counts	*Singles (*%)
**1**	156	100%	2,716	99.8%
**2**	243	100%	276	98.9%
**3**	49	100%	493	100%
**5**	106	99.1%	281	100%
**7**	594	100%	1,621	95.6%
**8**	34	100%	52	100%
**Total**	**1,182**	**99.9%**	**5,439**	**98.5%**

The single target sequences that constitute these *Doubles* given as “*Singles* Counts” and “*Singles* %” (percent of total single target sequences tabulated in Section A in Table 1).

**Fig 3:**
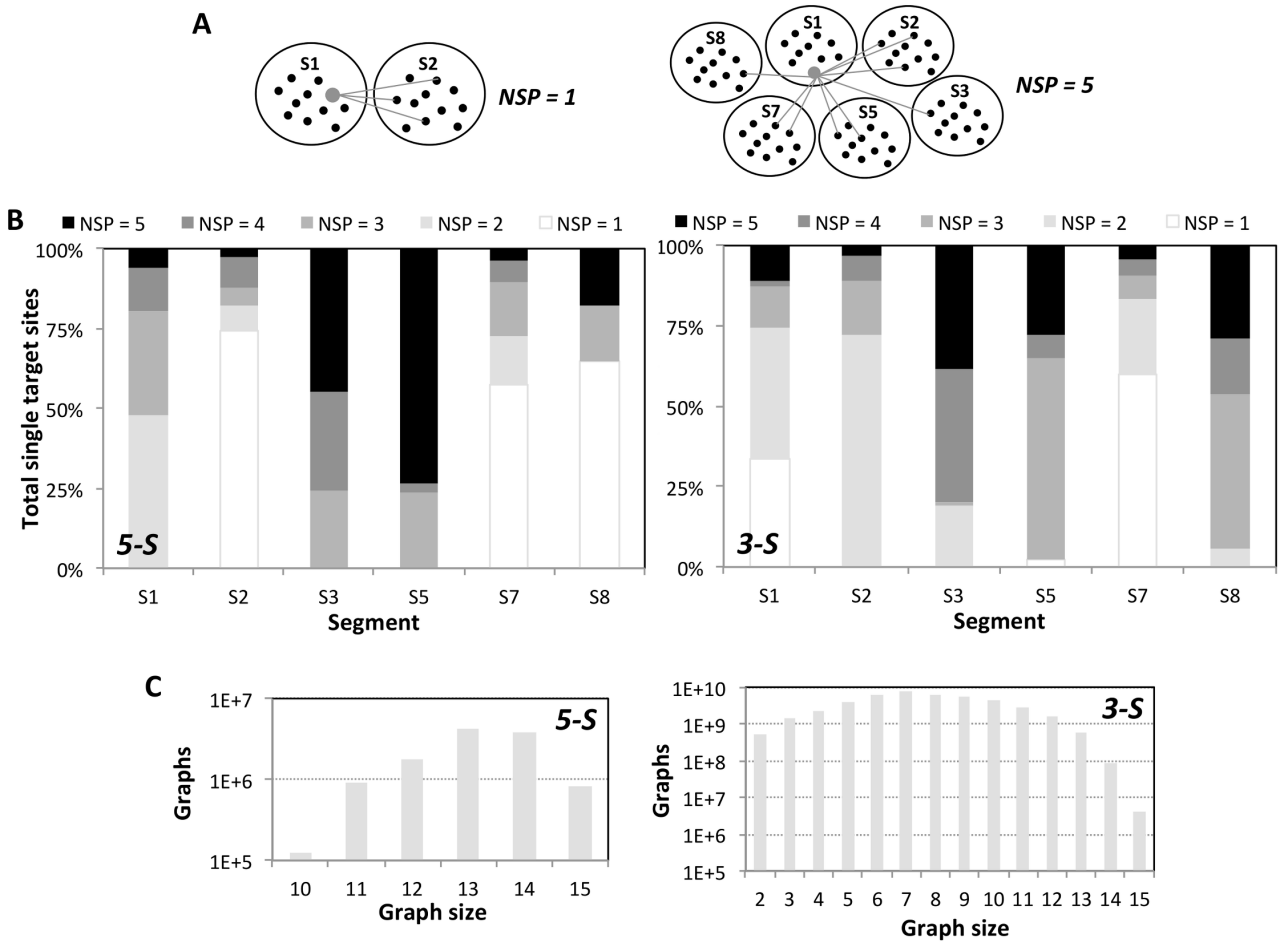
*NSP* of single target sequences in an effective *Double and* segment partner graphs. **(A) Schematic to determine the *NSP***. An effective *Double* is represented by a grey line connecting a pair of nodes denoting single target sequences from two target segments. For illustration, the *NSP* of a single target sequence in segment 1 (depicted as an enlarged grey node) is unity in the left panel (as it forms an effective *Double* only with segment 2 target sequences) and five in the right panel (as it forms an effective *Double* with target sequences from each of the other five segments). **(B) *NSP* frequency distributions**. Number of single target sequences against *NSP* by target segment in 5-S (left) and 3-S (right) sets plotted as 100% stacked bar charts. **(C) 6-vertices *(NSP = 5)* segment partner graphs.** The size (number of effective *Doubles*) distribution of all permutations of 6-vertices segment partner graphs constructed by single target sequences with *NSP = 5* from the six internal segments were plotted in absolute number of graphs for 5-S (left) and 3-S (right).

For every single target sequence tallied in Table 3, the number of segment partners (*NSP*) was determined by counting the number of segments with which it can form an effective *Double* with their respective single target sequences; *NSP* thus ranges from one to five, as illustrated in Fig 3A. Interestingly, the frequency distributions of *NSP* in each segment show that the number of target sequences with a high *NSP* is common and in some segments, the minimum *NSP* of all single target sequences is greater than unity (Figs 3B and S9A). This indicates a high reusability of a target sequence to form effective *Doubles* with different target segments.

As there are single target sequences in every segment whose *NSP* is five (Fig 3B), we investigate the size distribution of all 6-vertices segment partner graph formed by one (*NSP = 5*) target sequence from each of the six internal segments. The graph size is the sum of edges with each edge connecting two vertices (representing target sequences) denoting an effective *Double*; hence, the size indicates the number of effective *Doubles* in a particular segment partner graph. As shown in Figs 3C and S9B, the modal number of effective *Doubles* per graph in 5-S and 3-S is 13 and 7 respectively, and every graph in 5-S has at least 10 effective *Doubles*. In addition, 808,704 and 3,944,376 graphs in 5-S and 3-S respectively have 15 effective *Doubles*, which are termed as complete graphs i.e., every pair of distinct vertices is connected by a unique edge (S9B Fig). Identical analyses of 6-vertices segment partner graphs constructed by single target sequences with *NSP ≥ 1* (5-S) and with *NSP ≥ 4* (3-S) also reveal significant number of graphs with a big size (≤ 14) (S10 Fig). The existence of big-sized and complete graphs creates the possibility to hedge against antiviral resistance, as described next.

### Hedging against antiviral resistance

**Fig 4:**
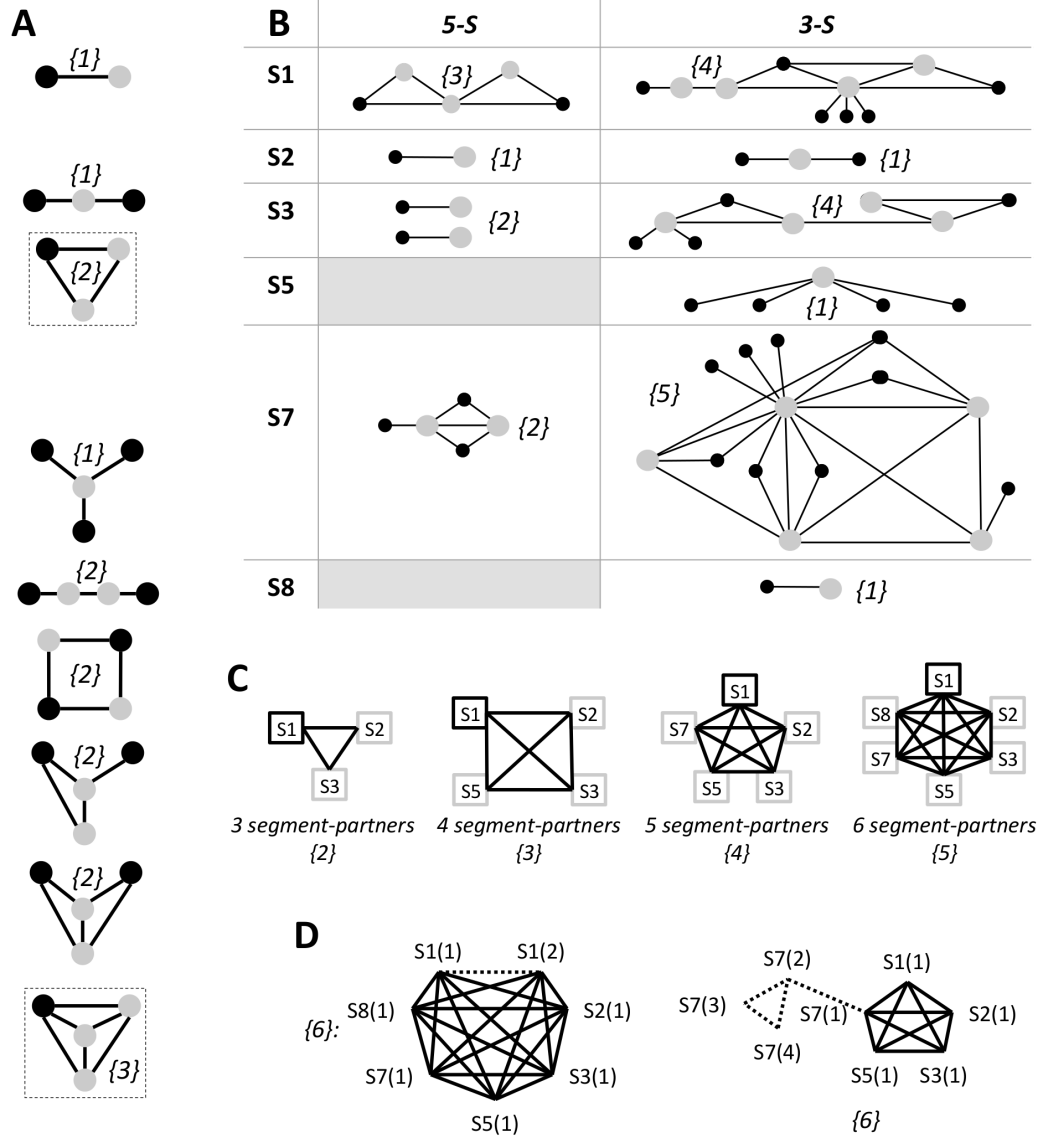
Hedge-factor of a set of target sequences. Each graph depicts a set of selected target sequences (shown as nodes) wherein an edge represents an effective *Dual* or *Double*. The nodes used to compute the hedge-factor (given in brackets) are either shaded or bordered grey. **(A) Representative values of hedge-factor, *HF*.** *HF* of all possible 2-, 3- and 4-vertices graphs are shown. Complete graphs are demarcated by a border. **(B) Maximum *HF* of effective *Duals* clusters.** The topologies of the *Duals* clusters from Fig 2 and their maximal *HFs* were presented. **(C) Maximum *HF* of segment partner graphs by effective *Doubles*.** As each internal segment can be used to form complete graphs exhibiting a range of *HF* from 2 to 5, only representative complete graphs are shown. **(D) Representative target sequence graphs with *HF = 6* constructed by combining effective *Duals* and effective *Doubles*.** Effective *Duals* and effective *Doubles* are denoted by broken and full edges, respectively. The running number in the round bracket of a target sequence’s label is purely schematic to denote different target sequences.

To mitigate antiviral resistance, a minimum of three target sequences is needed so that there is a target sequence to replace one that has become resistant. In this context, a resistant target sequence is unable to achieve 100% coverage of all subtypes strains when paired with another non-resistant target sequence. We define the hedge-factor to evaluate the extent a set of target sequences can potentially mitigate antiviral resistance. When depicted in a graph, wherein vertices denote selected target sequences and an edge represents an effective *Dual* or *Double*, the hedge-factor is the minimum number of resistant target sequences that will eliminate all the edges therein i.e. abolish the set’s therapeutic effectiveness to achieve 100% coverage. The hedge-factor in a set is maximal when the target sequences form a complete graph (demarcated with a border in Fig 4A) i.e. every pair of distinct vertices is connected by a unique edge; in an *n*-vertices complete graph, maximum hedge-factor *is n– 1* for *n ≥ 3*. A set of target sequences that forms a complete graph has the advantage of maintaining the 100% coverage of all strains by any pair of target sequences, which is valuable when target sequences need to be interchanged upon developing resistance.

For effective *Duals*, Fig 4B gives the maximum hedge-factor (number of grey nodes) in each target segment. Complete graphs, which are mostly 3-vertices graphs, are formed only in segments 1, 3 or 7 –S1 (5-S: two *3*-vertices; 3-S: three *3*-vertices), S3 (3-S: two *3*-vertices) and S7 (S-5: two *3*-vertices; 3-S: one *4*-vertices and seven *3*-vertices). Generally, complete graphs in 5-S have higher hedge-factors than in 3-S, and the highest hedge-factor of five is attained in segment 7. In fact, although the number of effective *Duals* in segment 7 is lowest among the three target segments in 3-S (Section B in Table 1), they form the biggest and most number of complete graphs. For effective *Doubles*, complete graphs involving 3, 4, 5 or 6 segments resulting in hedge-factors from two to five exist (Fig 4C). Myriad combinations of effective *Doubles* and effective *Duals* can be used to construct target sequence graphs that surpass the hedge-factor limit of five from using *Doubles* or *Duals* alone, two of which are depicted in Fig 4D.

**Fig 5:**
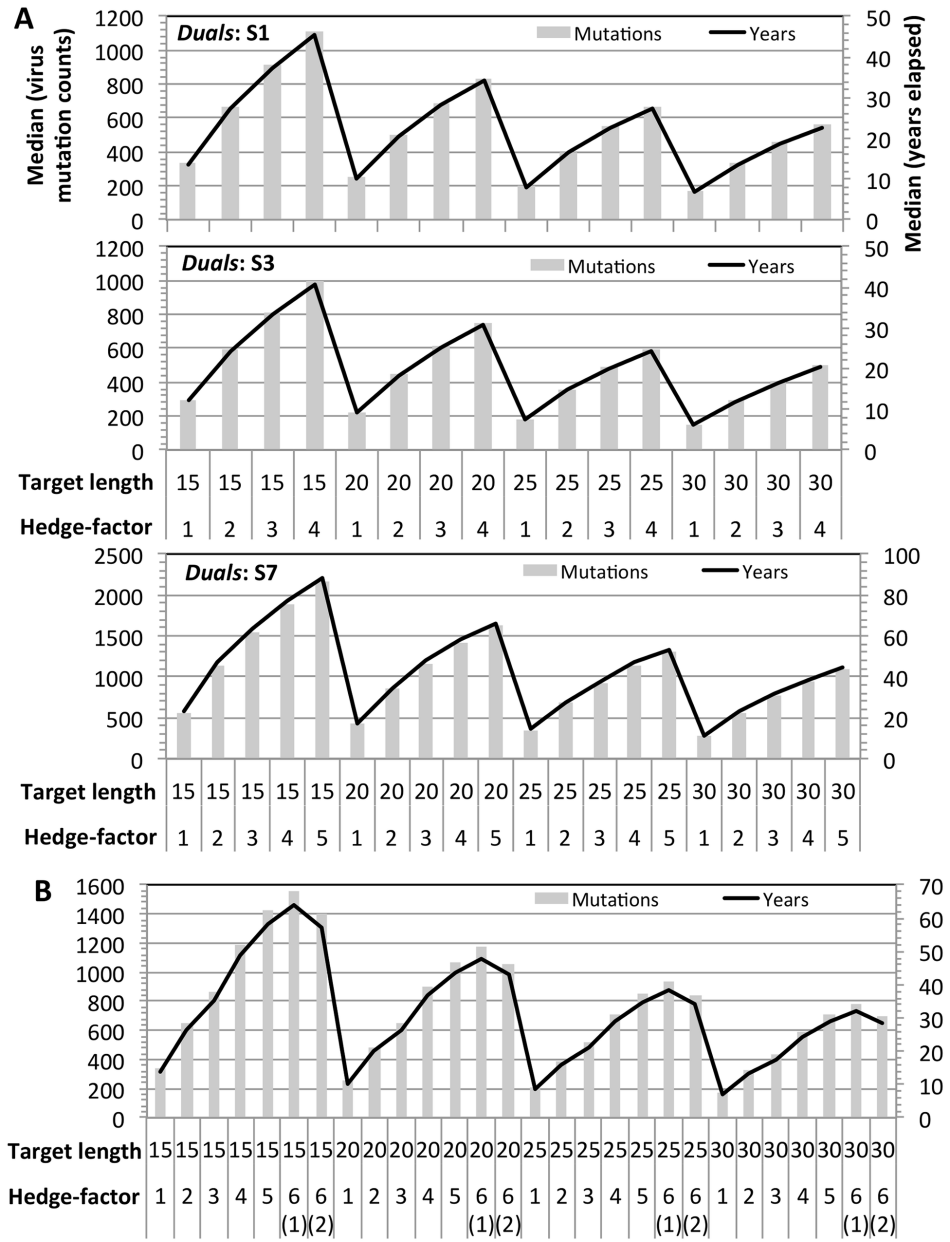
Mutation counts and time to resistance. For each set of target sequences, various sequence lengths ranging from 15 to 30 nucleotides were considered, and for simplicity, the length of all the target sequences in a set is identical. The median mutation counts and median time to resistance were obtained from 100,000 independent Monte Carlo simulations. **(A) Sets of effective *Duals*.** Only effective *Duals* from segments 1, 3 and 7 were used in the simulations as they are able to form complete graphs with *HF ≥ 2*. **(B) Sets of effective *Doubles* and sets of effective *Duals and effective Doubles combined*.** For *2 ≤ HF ≤ 5*, complete graphs formed by effective *Doubles* depicted in Fig 4C were used in the simulations. For *HF = 1*, effective *Doubles* targeting segments 1 and 2 were used. For *HF = 6*, the target sequence graphs that combine effective *Doubles* and effective *Duals* in Fig 4D were used; 6(1) and 6(2) respectively denote the left and right graphs in Fig 4D.

Monte Carlo simulations were used to study the effect of hedge-factor on the mutation counts and time elapsed for a set of *n* target sequences to become resistant i.e. lose its 100% coverage. In the simulation model (Materials and Methods and S2 Text), it is considered resistant when *n– 1* target sequences are resistant; a target sequence becomes resistant when its target site acquires a mutation and thereby cannot achieve 100% coverage. Thus, the mutation count is the minimum number of mutations in a virus to become resistant to a set of target sequences, with the corresponding time elapsed obtained by dividing the mutation counts with the average total annual mutation events in a virus [45]. Mutation events in 100,000 viruses were simulated for each set of target sequences to determine the median mutation counts and median time to resistance. Fig 5A plots the medians for sets of effective *Duals* forming complete graphs for a range of hedge-factors in segments 1, 3 and 7 depicted in Fig 4B. Expectedly, as the hedge-factor of a set is increased, substantially more mutation events and longer time is required to attain resistance. Likewise, complete graphs of effective *Doubles* in Fig 4C with higher hedge-factors possess considerably larger capacity to hedge against resistance (Fig 5B). Lastly, the target sequence graphs that combine effective *Doubles* and effective *Duals* in Fig 4D to augment the hedge-factor can further increase their hedging capacities (*HF = 6(1)* in Fig 5B).

### Cross-reactivity of the target sequences in human, pig and chicken hosts

The target sequences were aligned with up to one mismatch to the genomes and transcriptomes from human, pig and chicken hosts (Materials and Methods). Up to 4.7% and 3% of them in 5-S and 3-S sets respectively were found in one or more of the hosts’ transcriptomes whereas up to 22% and 12.7% of target sequences in 5-S and 3-S sets respectively were found in one or more of the hosts’ genomes (Section A of S4 Table). The 56 and 165 respective target sequences in 5-S and 3-S sets that hit the human transcriptome were mapped to 36 and 133 human genes (Section B of S4 Table). Among the 27 and 89 respective genes whose expression data were known, which correspond to 45 and 122 hit target sequences in 5-S and 3-S sets respectively, not all are expressed in tissues of the respiratory system. Nevertheless, when all the hit target sequences from each set independent of their tissue expression status were removed, complete heterosubtypic coverages by either effective *Duals* or effective *Doubles* (Tables 4 and 5), as well as maximal hedging against resistance by complete graphs (S11A Fig) are retained; all the data are made available at S1 Text. Similarly, when the respective target sequences in 5-S and 3-S sets that hit either the human genome or transcriptome were excluded, complete heterosubtypic coverages by effective *Duals* or *Doubles*, and hedging against resistance by complete graphs are conserved (Tables 4 and 5, S11B Fig and S1 Text).

**Table 4:** Complete heterosubtypic coverage by effective *Duals* and effective *Doubles* in the absence of target sequences that hit the human transcriptome or genome.

**A**		
**Target sequences**	***5-S***	***3-S***
**Segment**	**Counts**	**Counts**
**1**	153 | 131	2,691 | 2,552
**2**	223 | 156	250 | 164
**3**	43 | 10	480 | 395
**5**	102 | 77	228 | 127
**7**	573 | 522	1,660 | 1,549
**8**	33 | 27	49 | 28
**Total**	**1,127 | 923**	**5,358 | 4,815**
**B**		
**Effective *Duals***	***5-S***	***3-S***
**Segment**	**Counts**	**Counts**
**1**	493 | 130	11,659 | 5,228
**2**	25 | 0	30 | 0
**3**	59 | 6	6,386 | 3,768
**5**	–	2,316 | 298
**7**	506 | 320	1,987 | 808
**8**	–	2 | 0
**Total**	**1,083 | 456**	**22,380 | 10,102**

**(A)** Breakdown of target sequence counts by segment. **(B)** Number of effective *Duals*.

**Table 5:** Complete heterosubtypic coverage by effective *Doubles* in the absence of target sequences that hit the human transcriptome or genome.

	*5-S*					*3-S*				
Segment	2	3	5	7	8	2	3	5	7	8
**1**	297 | 16	441 | 94	6,041 | 2,966	3,945 | 1,496	93 | 0	6,081 | 1,207	98,235 | 64,580	29,024 | 12,854	51,901 | 23,833	4,173 | 1,492
**2**	–	286 | 12	1,957 | 267	2,075 | 549	55 | 0	–	3,378 | 399	3,846 | 561	3,026 | 748	125 | 0
**3**	–	–	3,047 | 473	574 | 76	67 | 12	–	–	25,204 | 11,530	8,304 | 3,094	1,964 | 678
**5**	–	–	–	1,908 | 953	370 | 0	–	–	–	4,940 | 1,966	1,110 | 49
**7**	–	–	–	–	962 | 639	–	–	–	–	1,492 | 641
**Total**	**22,118 | 7,553**					**242,803 | 123,632**				

Counts obtained without hits to the human transcriptome are given before “|” whereas counts obtained without hits to the human genome and transcriptome are given after “|”.

## Discussion

The capacity to drug most RNAs motivates the investigation on viral RNA targeting to address multiple circulating human subtypes and to mitigate antiviral resistance. 123,060 segment sequences and 35,938 virus strains from H1N1 (prior 2009), PD09 (2009 pandemic H1N1pdm09), H3N2, H5N1 and H7N9 representing the most prevalent human infecting subtypes over the past four decades were used to identify and characterize two sets of target sequences, each with a minimum length of 15 bases, for their coverage in targeting the multiple subtypes either singly or in pairs. A total of 1,183 conserved target sequences in the 5-S set and 5,523 conserved target sequences in the 3-S set (only H1N1, PD09 and H3N2 subtypes were analysed) were identified in all but segments 4 and 6 (Section A in Table 1). Notably, simultaneous heterosubtypic targeting of all the subtypes is achieved when specific pairs of same-segment (effective “*Duals*”) or two-segment (effective “*Doubles*”) target sequences are used. In 5-S and 3-S respectively, large numbers of effective *Duals* (1,662 and 29,124) and effective *Doubles* (28,463 and 280,351) exist (Section B in Table 1 and Table 2). The target selection space of *Doubles* is larger (Section B in Table 1 vs. Table 2)–(a) there are about 10 times more effective *Doubles* than effective *Duals*; (b) almost all single target sites can be paired to form effective *Doubles* but not effective *Duals*.

The specific pairings of multiple effective *Duals*, effective *Doubles* or both can generate distinct sets of target sequences each with different potential to hedge against antiviral resistance, as indicated by its hedge-factor (Fig 4). As the hedge-factor is maximal when target sequences in a set form a complete graph, they would be top choices for target selections. The number of possible complete graphs is enormous particularly those formed among effective *Doubles* (Figs 3C and S9B). Importantly, because effective *Doubles* in the six internal segments can form complete graphs (Fig 4C vs. Fig 4B) unlike effective *Duals* where complete graphs exist only in three segments (1, 3 and 7), they can hedge against resistance arising from segment reassortment events. That is, when a target segment undergoes reassortment and thereby becomes resistant to a target sequence, there are options to target another segment. Multi-segment targeting could be essential to address the observation that a third of avian flu A virus samples harbours at least one reassorted segment [46–47], although the reassortment frequency is likely lower in human subtypes. The Monte Carlo simulation results corroborate the use of combinatorial targets to significantly prolong antiviral resistance in HIV infections to a timescale typical for a chronic disease [48]. Incidentally, several target sequences in 5-S and 3-S sets that have been experimentally validated to reduce virus titre can be paired to form effective *Duals*, effective *Doubles*, and target graphs with hedge-factor of 2 (S3 Text). Thus, they can be readily be used for animal studies prior human clinical trials.

Although the time to resistance of a set of target sequence is primarily dependent on the hedge-factor, it is affected by target sequence length and target segment mutation rate (Fig 5). At a given hedge-factor, the time to resistance correlates inversely with target sequence length since a long target sequence has more nucleotides to acquire a mutation. Target sequences in a relatively slower mutating segment have longer time to resistance–for instance, sets of effective *Duals* in segment 7 has the longest time to resistance for the same hedge-factor and target sequence length (Fig 5A). In summary, Fig 5 provides a reference for specifying key parameters pertaining to target segment(s), hedge-factors, target sequence length, and hedging capacity. Together with Fig 4, they offer the toolkits for assembling sets of target sequence for specific therapeutic objectives.

Besides efficacy and efficiency, off-target side effects are major obstacles to a successful drug in human studies. In the context of viral RNA suppression as a therapeutic strategy, off-target effects occur when a viral target site is also found in the host cell transcriptome whose expression is essential. Notably, when target sequences with potential off-target effects were removed, the reduced numbers of effective *Duals*, effective *Doubles* and complete graphs still remain enormous as drug targeting space; for example, the 3-S set has 22,380 effective *Duals*, 242,803 effective *Doubles*, and 905,850 complete graphs of size 15 (Tables 4 and 5 and S11 Fig). Other off-target effects result from polypharmacological properties of a drug through binding to unspecific target sites, high-dose non-specificity effects, immunological response [49] or other non-sequence dependent effects [43,50]. Specific nucleic acid chemistry and modifications such as morpholino and 2’-O-methyl with phosphorothioate or phosphorodiamidate backbones have improved binding specificity [43,51–53] and could help to overcome some of these problems.

Due to the relative small number of available H5N1 and H7N9 strains (S1 Table), they were excluded in the determination of conserved target sequences in the 3-S set. Consequently, the number of target sequences, effective *Duals* and effective *Doubles* in 3-S is about an order of magnitude larger than in 5-S (Table 1); an expanded target space is typically useful for therapeutic development. Notably, consideration of H5N1 and H7N9 subtypes does not affect the coverage of target sequences against human subtypes, as the coverage distributions against H1N1, PD09, H3N2 and *H00N00* in both sets are similar (S12 Fig). In contrast, coverage of target sequences in the 5-S set was greatly improved over in the 3-S set against all animal subtypes as well as both *zoonotic* and *exotic* groups of animal subtypes (S12 Fig), except for segments 2 and 8. Thus, one can select target sequences in segments 2 and 8 from the 3-S set for their larger target space and target sequences in segments 1, 3 5 and 7 from the 5-S set for their better extensibility against cross-species subtypes to pre-empt future strains that cross from animal to human hosts.

Curated strains with incomplete genomes and segment sequences with non-full-length were both included in the analyses for considering as many sequence variations as possible. When only complete-genome strains were analysed for both 5-S and 3-S sets, the number of target sequence pairs and their combinations do not change significantly. This is expected because the analyses of both effective *Duals* and effective *Doubles* do not require genome completeness. Moreover, all the effective *Duals*, effective *Doubles* and complete graphs of size 15 from the all-genome analysis are complete subsets of those in the complete-genome analysis (S5 Table). On the other hand, when only full-length segment sequences were analysed, significantly more target sequence pairs and their combinations were obtained in both 5-S and 3-S sets. Similarly, all the effective *Duals*, effective *Doubles* and complete graphs of size 15 from the all-length analysis are complete subsets of those in the full-length analysis, except for five effective *Duals* (out of 29,124) and 54 effective *Doubles* (out of 280,351) in the 3-S set (S6 Table). Therefore, not all results from the complete-genome and full-length analyses respectively are applicable to complete- and incomplete-genome strains, and to full-length and non-full-length segments. In short, considering only complete genomes and full-length segments overestimates conservation of candidate sites while additional variations observed in incomplete genomes/segments lead to un-selection of more sites as not being conserved. Therefore, an analysis considering all available influenza sequences will provide the most robust selection hedging against further genomic changes and natural virus evolution.

This study inevitably poses the following questions–which segment to target when *Duals* are used, which segment combinations to target when *Doubles* are used, and how to prioritise a set of RNA drugs targeting multiple sequences for clinical trials? Reduction of virus titre upon either knockdown or knockout of specific segment have been reported [19–42], however, there is no comparative study to determine the relative suppression by each targeted segment on virus viability and replicability. Moreover, only four out of 15 combinations of double segments targeting have been reported; segment 5 paired with segment 1, 2, 3 or 7 [26,29,31–33]. The feasibility to target a segment is also dependent on the accessibility of its target sequence’s secondary structures for efficient drug binding. The secondary structures and thereby binding accessibility of a target sequence between subtypes can vary due to nucleotide variations among segment sequences (S13 Fig). To facilitate the selection of a set of target sequences that lead to efficient RNA therapeutics targeting viral RNA, mRNA or cRNA, the following resources for respective 5-S and 3-S sets are made available for download–coverages of all target sequence against all analysed strain sequences from human and animal subtypes, pairings of all effective *Duals* and *Doubles*, and binding accessibilities [54] of every target sequence (S1 Text); two versions (inclusion or exclusion of hit target sequences to the human transcriptome or genome) are provided per resource.

It is possible that RNA therapeutics could develop antiviral resistance easier than protein-targeting drugs via silent mutations. However, at least three simultaneous mutations at a target sequence are required to abrogate the AON efficiency [30,34], which further substantially increase the mutation counts and prolongs the elapsed time required for a set of target sequences to become resistant (S14 Fig). The availability of drugs for simultaneous heterosubtypic targeting is likely to be more effective and therefore may slow down and reduce the severity of pandemic and seasonal flu infections, which limits the reservoir of hosts for the virus to evolve. In addition, clinical administration of a drug cocktail with more than two RNA drugs to further delay antiviral resistance is worth exploring albeit two targets are theoretically sufficient to address all prevalent subtypes. Nonetheless, since few genomic modifications suffice to create a novel virus strain with strongly altered transmission phenotype [3–4], the strategy of selecting a resistance-hedging set of multiple target sequences is particularly relevant as some of the target sequences are likely to remain effective against a new strain. This is corroborated from the results that there are human target sequences that are found in more than 90% of the unique sequences from a total of 109 human and animal subtypes of differing zoonotic potential. (S3, S4, S5, and S6 Figs). Finally, the concept of *Duals*, *Doubles* and hedge-factor can potentially be applied to other viruses that manifest multiple subtypes to develop RNA therapeutics addressing the subtypes simultaneously and for mitigating antiviral resistance.

## Materials and Methods

### Sources of sequence data

Influenza A virus nucleotide sequences from both human and animal hosts were downloaded from GenBank for all eight segments for H1N1 (before 2009), PD09 (2009 pandemic H1N1), H3N2 and H5N1 subtypes. For the H7N9 subtype, nucleotide sequences for both human and animal hosts were downloaded from the Global Initiative on Sharing All Influenza Data (GISAID) Epiflu database; we acknowledge the authors, originating and submitting laboratories of the sequences analysed from the Database, listed in gisaid_acknowledge_table_processed.txt. To further assess the degree of conservation and extensibility of target sequences, three other groups of influenza viruses were downloaded from GenBank. The first group consists of viruses from any other subtypes with history of infecting humans: H1N2, H2N2, H6N1, H7N2, H7N3, H7N7, H9N2 and H10N8, collectively named as “*H00N00*”. The second group, named as “*zoonotic*”, is considered as having a higher zoonotic potential. To obtain this set of viruses, every protein from all viruses from the *H00N00* group was used as query to the tachyon server [55] and all animal strains that are within the top 50 hits with a tachyon score of 0.8 or more would be considered as having zoonotic potential. The third group of viruses, named as “*exotic*”, are viruses from less common host sources like Equine, Canine, Ferret, Cat, Seal, Tiger, Pika, Mink, Bat, Penguin, Bovine, Wild boar, Raccoon dog, Camel, Leopard, Muskrat, Cheetah, Feline, Stone marten, Panda, Civet, Whale, Giant anteater, Blow fly or Beetle origin. Sequences used in the analyses were downloaded on 29^th^ April 2014. To ensure that only unique sequences were analysed for each subtype, the redundant identical sequences were removed with Cd-hit [56] by allowing a maximal sequence identity of 100%. Although UTRs were not used in identifying target sequences, they were not removed from the sequences as they were used for secondary structure predictions when designing AONs.

### Monte Carlo simulations for estimating mutation counts and time to resistance

A computational model is developed to simulate the random single nucleotide substitution events in each of the eight viral segments; the model assumptions are discussed in S2 Text. Monte Carlo simulations were applied on the model as follows:

1. Specify the parameters of the target sequence set: target sequence length and target segments.
2. 9 independent random number generators (*R*_*0*_, *R*_*1*_, *R*_*2*_, *R*_*3*_, *R*_*4*_, *R*_*5*_, *R*_*6*_, *R*_*7*_ and *R*_*8*_) are initialized.
3. A random number is generated from *R*_*0*_ to determine the next segment where the next nucleotide substitution event will occur. The probability of each segment where a substitution will occur is estimated from reported segment mutation rates (see below).
4. Given the next segment where the substitution will occur, the corresponding random number generator *R*_*i*_ is used to determine the nucleotide position in the segment where the substitution occurs.
5. Increment the mutation counts by one.
6. Check the resistance status of every target sequence. Repeat steps 3 to 5 until the set becomes resistant.
7. Output the mutation counts and compute the time elapsed (see below).
8. Repeat steps 2 to 7 for a total of 100,000 runs.
9. Determine the medians of the mutation counts and time to resistance from the 100,000 runs.

The segment mutation rates (S2 Text) are used to compute the probability of each segment, *Pr(Segment)*, where the next substitution mutation will occur in the Monto Carlo simulations, and the time to resistance. *Pr(Segment)* is calculated by normalizing the segment’s mutation rate with the total number of substitution events from all the segments in a year. Lastly, the time to resistance is computed by dividing the mutation counts with the total number of substitution events from all the segments in a year. The simulation was implemented in Java^Tm^ programming language; source codes can be downloaded at http://mendel.bii.a-star.edu.sg/SEQUENCES/HEDGING_DRUG_RESISTANCE/source-codes/index.html.

### Cross-reactivity of the viral target sequences in human, pig and chicken hosts

The human (GCF_000001405.28_GRCh38.p2), pig (GCF_000003025.5_Sscrofa10.2) and chicken (GCF_000002315.3_Gallus_gallus-4.0) genomic and transcriptomic sequences were downloaded from the NCBI genome resource (ftp://ftp.ncbi.nlm.nih.gov/genomes/all/). The *blastn* program [57] was used with both the default parameters, and also with parameters adjusted for short sequence searches (e-value of 1000, word size 7, no complexity masking) to search the target consensus sequences against the human transcriptome and genome for possible cross-reactivity. In order to reduce cross-reactivity, target sequences that have a hit in the human transcriptome were removed. The hits had maximally one mismatch and no target sequence was found to have more than one mismatch from the above search criteria. In any case, sequences with two- and three-mismatches are inefficient and ineffective to target respectively [30,34]. The genes in the human transcriptome that match the target sequences for up to one mismatch were examined for their gene expression profile in 84 tissue types using data obtained from Gene Atlas (Human U133A/GNF1H, GSE1133, http://biogps.org/downloads, click the link to gnf1h-gcrma.zip) [58]. Perl scripts were used to map the accessions from the human ptome to their respective genes using the NCBI gene2accession data file (ftp://ftp.ncbi.nlm.nih.gov/gene/DATA/gene2accession.gz); the scripts can be downloaded at http://mendel.bii.a-star.edu.sg/SEQUENCES/HEDGING_DRUG_RESISTANCE/source-codes/index.html.

## Supporting Information

S1 Table Breakdown on sequence counts and strains of H1N1, PD09, H3N2, H5N1 and H7N9. The total sequence counts in the curated database used to determine the unique sequences are given in parentheses.
(DOCX)

S2 Table Breakdown of number of unique segment sequences and strains from aH1N1, aH3N2, aH5N1 and aH7N9 subtypes and from *H00N00*, *zoonotic* and *exotic* groups of subtypes. The total sequence counts in the curated database used to determine the unique sequences are given in parentheses.
(DOCX)

S3 Table Number of virus strains analysed in pairing of target segments. Available human infecting virus strains and their segment sequences from H1N1, PD09, H3N2, H5N1 and H7N9 were downloaded from GenBank and Global Initiative on Sharing All Influenza Data (GISAID) Epiflu^TM^ databases. The quantity in a cell indicates the total number of virus strains in which both of their targeted segment sequences are available from either five subtypes in **(A)** 5-S set or from three subtypes (H1N1, PD09 and H3N2) in **(B)** 3-S set.
(DOCX)

S4 Table Hit target sequences in the transcriptomes and genomes from human, pig and chicken hosts. **(A)** Number of target sequences in 5-S and 3-S sets that were found (up to one mismatch) in the transcriptomes and genomes of human, pig and chicken hosts. The % column tabulates the percentage of hit target sequences in the total target sequence in each set. **(B)** Number of human genes (with and without expression data) that were mapped from the accessions for which the viral target sequences was found.
(DOCX)

S5 Table Complete heterosubtypic coverage and resistance hedging when only viral strains with complete-genome were analysed. 11.5% of the total unique internal segment sequences that do not belong to complete genomes were removed. Counts from the all-genome analysis are given in parentheses for comparison. **(A)** Target sequences. No target sequence can achieve 100% heterosubtypic coverage in the all-genome analysis. * Except for 15 new target sequences in segment 7 in the 3-S set, the target sequences in both 5-S and 3-S sets are identical in both all- and complete-genome analyses. **(B)** Effective *Duals*. All effective *Duals* from the all-genome analysis are complete subset of those from the complete-genome analysis. **(C)** Effective *Doubles*. All effective *Doubles* from the all-genome analysis are complete subset of those from the complete-genome analysis. **(D)** Size distribution of all 6-vertices segment partner graphs formed by a target sequence (whose *NSP = 5*) from each of the six internal segments (Figs 3C and S9B). * Complete graphs of size 15.
(DOCX)

S6 Table Complete heterosubtypic coverage and resistance hedging when only full-length viral segment sequences were analysed. 11% of the total unique internal segment sequences that are non-full-length were removed. Counts from the all-length segment sequence analysis are given in parentheses for comparison. **(A)** Target sequences. No target sequence can achieve 100% heterosubtypic coverage in the all-genome analysis. No single target sequence can achieve 100% heterosubtypic coverage in the full-length analysis. * Except for one target sequence in segment 7 in the 3-S set, the target sequences in both 5-S and 3-S sets are identical in both full- and all-length analyses. **(B)** Effective *Duals*. * Except for five effective *Duals* in segment 7 in the 3-S set, all effective *Duals* from the all-length analysis are complete subset of those from the full-length analysis. ** 21 effective *Duals* in segment 5 were obtained in the full-length analysis whereas there was none in the all-length analysis. **(C)** Effective *Doubles*. * Except for six S1-S7, 45 S5-S7, and three S7-S8 effective *Doubles* in the 3-S set, all effective *Doubles* from the all-length analysis are complete subset of those from the full-length analysis. **(D)** Size distribution of all 6-vertices segment partner graphs formed by a target sequence (whose *NSP = 5*) from each of the six internal segments (Figs 3C and S9B). *Complete graphs of size 15.
(DOCX)

S1 Text Description of data resources. (DOCX)

S2 Text Monte Carlo simulations for estimating mutation counts and time to resistance. (DOCX)

S3 Text Validated target sequences. (DOCX)

S1 Fig Cumulative frequencies of the 5-S set target sequences coverage by target segments. In the graphs, each data point denotes the cumulative number of target sequences (vertical axis) in a particular target segment with a minimum coverage (horizontal axis). The coverage of a target sequence is defined as the percentage of unique segment sequences in the corresponding target segment from subtypes H1N1 (grey), PD09 (orange), H3N2 (red), H5N1 (green), H7N9 (blue) and all the five subtypes (black) in which a match with the target sequence was found. In the occasional incident that the target site of a unique sequence contains an ambiguous base, it is processed by the following rules. The unique sequence is not considered a match when all possible bases of its ambiguous base do not match the respective base of the target sequence; for instance, a K (denotes either G or T) ambiguity code is found at the unique sequence where it is a C at the corresponding target sequence. Otherwise, the unique sequence is omitted during the computation of target sequence coverage when one of the possible bases of its ambiguous base matches the respective base of the target sequence (i.e. the unique sequence is neither a match nor a no-match; for instance, a K (denotes either G or T) ambiguity code is found at the unique sequence where it is a T at the corresponding target sequence.
(TIF)

S2 Fig Cumulative frequencies of the 3-S set target sequences coverage by target segments. Refer to S1 Fig legend. In this case, only unique sequences from 3 subtypes (H1N1, PD09 and H3N2) were used.
(TIF)

S3 Fig Coverage of target sequences in other human and animal subtypes. Coverage of each target sequence against subtypes H1N1, PD09, H3N2, H5N1, H7N9, aH1N1, aH3N2, aH5N1 and aH7N9, and against a collection of subtypes grouped as *H00N00*, *zoonotic* and *exotic* (refer to Materials and Methods in the main paper); refer to S1 Fig legend on the procedure to determine the coverage. For plotting purposes (left panel: 5-S; right panel: 3-S), all the target sequences in a segment were numbered (horizontal axis) after they were sorted ascendingly by their coordinates in the target segment followed by their target sequence length. **(A) Segment 1. (B) Segment 2. (C) Segment 3. (D) Segment 5. (E) Segment 7. (F) Segment 8.**
(TIF)

S4 Fig Cumulative frequencies of target sequences coverage in other human and animal subtypes. In the graphs (left panel: 5-S; right panel: 3-S), each data point denotes the cumulative number of target sequences (vertical axis) in a particular target segment with a minimum coverage (horizontal axis). Coverage of each target sequence against subtypes H1N1, PD09, H3N2, H5N1, H7N9, aH1N1, aH3N2, aH5N1 and aH7N9, and against a collection of subtypes grouped as *H00N00*, *zoonotic* and *exotic* (refer to Materials and Methods in the main paper); refer to S1 Fig legend on the procedure to determine the coverage. **(A) Segment 1. (B) Segment 2. (C) Segment 3. (D) Segment 5. (E) Segment 7. (F) Segment 8.**
(TIF)

S5 Fig Comparing target sequences coverage distributions in human (H1N1, PD09, H3N2, H5N1 and H7N9) and corresponding animal (aH1N1, aH3N2, aH5N1 and aH7N9) subtypes and in *H00N00* group of human subtypes. Differences in the coverage distribution of target sequences in each human subtype and in its corresponding animal subtype or in the *H00N00* group were tested for statistical significance. Coverage distributions of target sequences in different subtypes were compared by boxplots (vertical axis) and *student-t* test for 5-S (left) and 3-S (right) sets. One-sided *student-t* test was performed on the target sequences coverages against each human subtype and against its corresponding animal subtype (i.e. H1N1 vs. aH1N1, PD09 vs. aH1N1, H3N2 vs. aH3N2, H5N1 vs. aH5N1, and H7N9 vs. aH7N9), and against every human subtype and against the *H00N00* group of human subtypes (i.e. H1N1 vs. *H00N00*, PD09 vs. *H00N00*, H3N2 vs. *H00N00*, H5N1 vs. *H00N00*, and H7N9 vs. *H00N00*). Differences between two coverage distributions were considered as statistically significant when *p-value ≤ 0*.*001*. * denotes coverage distribution in the human subtype and the corresponding animal subtype is different (black: coverage distribution in the human subtype is statistically higher; red: coverage distribution in the animal subtype is statistically higher). # denotes coverage distribution in the human subtype and *H00N00* group is different (black: coverage distribution in the human subtype is statistically higher; red: coverage distribution in the *H00N00* group is statistically higher). Except for segment 5, coverage distribution in the human subtype is not always the highest. Particularly in the 5-S set, more incidences where coverage distribution in the human subtype is either similar to or lower than its corresponding animal subtype or the *H00N00* group are observed.
(TIF)

S6 Fig Comparing target sequences coverage distributions in animal subtypes (aH1N1, aH3N2, aH5N1 and aH7N9) subtypes and in *zoonotic* and *exotic* groups of animal subtypes. Differences in the coverage distribution in each human corresponding animal subtype and in the *zoonotic* or *exotic* groups of animal subtypes were tested. Coverage distributions of target sequences in different subtypes were compared by boxplots (vertical axis) and *student-t* test for 5-S (left) and 3-S (right) sets. One-sided *student-t* test was performed on the target sequences coverages against every animal subtype and against the *zoonotic* group of animal subtypes (i.e. aH1N1 vs. *zoonotic*, aH3N2 vs. *zoonotic*, aH5N1 vs. *zoonotic*, and aH7N9 vs. *zoonotic*), and against every animal subtype and against the *exotic* group of animal subtypes (i.e. aH1N1 vs. *exotic*, aH3N2 vs. *exotic*, aH5N1 vs. *exotic*, and aH7N9 vs. *exotic*), and against the *zoonotic* and *exotic* groups. Differences between two coverage distributions were considered as statistically significant for *p-value ≤ 0*.*001*. * denotes coverage distribution in the animal subtype and *zoonotic* group is different (black: coverage distribution in the animal subtype is statistically higher; red: coverage distribution in the *zoonotic* group is statistically higher). # denotes coverage distribution in the animal subtype or *zoonotic* group and *exotic* group is different (black: coverage distribution in the animal subtype or *zoonotic* group is statistically higher; red: coverage distribution in the *exotic* group is statistically higher). More incidences where coverage distribution in the human corresponding animal subtype is either similar to or lower than in the *zoonotic* group are observed for the 5-S set. Coverage distribution in the *exotic* group is generally the lowest.
(TIF)

S7 Fig Distribution of the target sequence positions from the effective *Duals* in each segment. Effective *Duals* in both 5-S and 3-S sets refer to pairs of single target sequences that can cover all unique sequences of respective target segments. No effective *Dual* was found for segments 5 and 8 of the 5-S set.
(TIF)

S8 Fig Collective distribution of the target sequence positions from effective *Doubles* in each segment. Effective *Doubles* in both 5-S and 3-S sets refer to pairs of single target sequences in different segment that can cover all virus strains. An effective *Double* is considered to cover a virus strain when one or both of its target sequences is found in either one or both of the virus strain’s targeted segment sequences. The target sequence position distribution depicted is aggregated from all effective *Doubles* target sequences obtained from all possible target segment pairings.
(TIF)

S9 Fig Frequency distributions of *NSP* and segment partner graphs. **(A) *NSP* frequency distributions**. Number of single target sequences against *NSP* by target segment in 5-S (top) and 3-S (bottom) sets plotted as bar charts. **(B) 6-vertices *(NSP = 5)* segment partner graphs.** The size (number of effective *Doubles*) distribution of all permutations of 6-vertices segment partner graph constructed by single target sequences with *NSP = 5* from the six internal segments were tabulated, and plotted in percentage of total graph permutations for 5-S (top) and 3-S (bottom) sets.
(TIF)

S10 Fig 6-vertices (*NSP ≥ 1*) segment partner graphs. The size (number of effective *Doubles*) distribution of all permutations of 6-vertices segment partner graph were tabulated (left), and plotted in absolute number of graphs (gray) and in percentage of total graph permutations (black). Graphs were constructed by single target sequences with **(A) *NSP ≥ 1* (5-S set)** and **(B) *NSP ≥ 4* (3-S set)** from the six target segments. Note: determination of graph size becomes computationally intractable in the 3-S set when single target sequences with *NSP ≤ 3* are considered, as a consequence of immense total graph permutations.
(TIF)

S11 Fig Size distribution of graphs formed by effective *Doubles* in the absence of target sequences that hit the human transcriptome or genome. **(A)** Size distribution of all 6-vertices segment partner graphs formed by a target sequence (whose *NSP = 5*) from each of the six internal segments (Fig 3C and S9B Fig) in 5-S (top panel) and 3-S (bottom) sets, after the removal of hits with the human transcriptome. The key results remain qualitatively unchanged–the modal number of effective *Doubles* per graph in 5-S and 3-S is 13 and 7 respectively, every graph in 5-S has at least 10 effective *Doubles*, and complete graphs (size = 15) that has the highest hedge-factor of five in both sets are still aplenty. **(B)** Upon removal of target sequences that hit the human genome or transcriptome, the size distribution of all 6-vertices segment partner graphs formed by a target sequence from each of the six internal segments in 5-S (column 1, *NSP* ≥ 1) and 3-S (columns 2 and 3, *NSP* ≥ 3 and *NSP* ≥ 2 respectively) sets. There are respectively 588 and 436,614 complete graphs of 5-vertices formed by a target sequence from S1, S2, S3, S5 and S7 (size = 10) in 5-S and 3-S sets.
(TIFF)

S12 Fig Comparing coverage distributions of target sequences between 5-S and 3-S sets. Coverage distributions of target sequences in H1N1, PD09, H3N2, *H00N00*, aH1N1, aH3N2, aH5N1, aH7N9, *zoonotic* and *exotic* were each plotted for 5-S and 3-S sets side-by-side.
(TIF)

S13 Fig Representative binding accessibilities of target sequences in all subtypes strains. Binding accessibilities of the two representative target sequences in every strain were computed (refer to S1 Text), and their distributions in each of the five human subtypes were depicted as boxplots. Due to variations in the segment sequence among the strains, differences in the segment mRNA *co-transcriptional* secondary structures can lead to different binding accessibility distributions (right) or have no considerable effect (left).
(TIF)

S14 Fig Mutation counts and time to resistance. The Monte Carlo simulations described in the main text and shown in Fig 5 were repeated with the condition that a target sequence is considered resistant when it acquires three substitution mutations (3-hits). For ease of comparison with Fig 5, the results labelled as “1-hit” were plotted together with 3-hits. Refer to Fig 5 legend.
(TIF)
