# Supplementary material for: Discovery of Influenza A Virus Sequence Pairs and Their Combinations for Simultaneous Heterosubtypic Targeting that Hedge against Antiviral Resistance
Source: PLoS Comput Biol. 2016 Jan 15;12(1):e1004663. doi: 10.1371/journal.pcbi.1004663 (PMC4714944; doi:10.1371/journal.pcbi.1004663)
Supplement: S1 Table — The total sequence counts in the curated database used to determine the unique sequences are given in parentheses. (DOCX) [file pcbi.1004663.s001.docx]

**Table S1. Breakdown on sequence counts and strains of H1N1, PD09, H3N2, H5N1 and H7N9**

| Segment | H1N1 | PD09 | H3N2 | H5N1 | H7N9 | Total |
| --- | --- | --- | --- | --- | --- | --- |
| 1 | 770  (968) | 2,782  (4,610) | 3,417  (4,820) | 170  (200) | 74  (100) | 7,213  (10,698) |
| 2 | 734  (991) | 2,736  (4,575) | 3,387  (4,832) | 167  (203) | 65  (95) | 7,089  (10,696) |
| 3 | 688  (976) | 2,640  (4,550) | 3,312  (4,807) | 166  (206) | 77  (95) | 6,883  (10,634) |
| 4 | 2,020  (2,917) | 7,781  (12,833) | 10,892  (16,244) | 303  (350) | 82  (123) | 21,078  (32,467) |
| 5 | 625  (989) | 2,021  (4,751) | 2,963  (5,107) | 164  (208) | 54  (95) | 5,827  (11,150) |
| 6 | 1,419  (2,031) | 4,387  (9,212) | 5,903  (8,565) | 265  (322) | 68  (120) | 12,042  (20,250) |
| 7 | 738  (1,534) | 1,724  (6,473) | 3,330  (7,595) | 151  (220) | 38  (105) | 5,981  (15,927) |
| 8 | 532  (1,007) | 1,745  (4,938) | 2,149  (4,986) | 131  (204) | 53  (103) | 4,610  (11,283) |
| Total | **7,526**  **(11,413)** | **25,816**  **(51,942)** | **35,353**  **(56,956)** | **1,517**  **(1,913)** | **511**  **(836)** | **70,723**  **(123,060)** |
| Strains | **3,486** | **14,198** | **17,743** | **379** | **132** | **35,938** |

The total sequence counts in the curated database used to determine the unique sequences are given in parentheses.
