# Supplementary material for: Discovery of Influenza A Virus Sequence Pairs and Their Combinations for Simultaneous Heterosubtypic Targeting that Hedge against Antiviral Resistance
Source: PLoS Comput Biol. 2016 Jan 15;12(1):e1004663. doi: 10.1371/journal.pcbi.1004663 (PMC4714944; doi:10.1371/journal.pcbi.1004663)
Supplement: S2 Table — The total sequence counts in the curated database used to determine the unique sequences are given in parentheses. (DOCX) [file pcbi.1004663.s002.docx]

**Table S2. Breakdown of number of unique segment sequences and strains from aH1N1, aH3N2, aH5N1 and aH7N9 subtypes and from *H00N00*, *zoonotic* and *exotic* groups of subtypes**

|  | Subtypes | S1 | S2 | S3 | S5 | S7 | S8 | Total | Strains |
| --- | --- | --- | --- | --- | --- | --- | --- | --- | --- |
| aH1N1 | 1 | 1,142  (1,362) | 1,158  (1,376) | 1,133  (1,374) | 1,152  (1,428) | 1,560  (2,285) | 1,077  (1,426) | **7,222**  **(9,251)** | **2,447** |
| aH3N2 | 1 | 796  (1,023) | 808  (1,024) | 798  (1,022) | 772  (1,052) | 903  (1,414) | 696  (1,065) | **4,773**  **(6.600)** | **1,513** |
| aH5N1 | 1 | 1,345  (1,619) | 1,316  (1,635) | 1,302  (1,596) | 1,215  (1,624) | 1,091  (1,743) | 1,157  (1,769) | **7,426**  **(9,986)** | **2,009** |
| aH7N9 | 1 | 81  (115) | 74  (114) | 75  (114) | 67  (118) | 63  (118) | 57  (119) | **417**  **(698)** | **123** |
| *H00N00* | 8 | 106  (139) | 112  (140) | 105  (132) | 118  (157) | 116  (162) | 112  (164) | **669**  **(894)** | **193** |
| *zoonotic* | 78 | 778  (1,061) | 1,227  (1,710) | 733  (963) | 1,584  (2,353) | 4,521  (7,215) | 2,086  (3,149) | **10,929**  **(16,451)** | **8,822** |
| *exotic* | 19 | 230  (262) | 243  (290) | 243  (306) | 222  (274) | 277  (452) | 310  (462) | **1,525**  **(2,046)** | **616** |
| Total |  | **4,478**  **(5,581)** | **4,938**  **(6,289)** | **4,389**  **(5,507)** | **5,130**  **(7,006)** | **8,531**  **(13,389)** | **5,495**  **(8,154)** | **32,961**  **(45,926)** | **15,723** |

The total sequence counts in the curated database used to determine the unique sequences are given in parentheses.
