# Supplementary material for: Discovery of Influenza A Virus Sequence Pairs and Their Combinations for Simultaneous Heterosubtypic Targeting that Hedge against Antiviral Resistance
Source: PLoS Comput Biol. 2016 Jan 15;12(1):e1004663. doi: 10.1371/journal.pcbi.1004663 (PMC4714944; doi:10.1371/journal.pcbi.1004663)
Supplement: S3 Table — Available human infecting virus strains and their segment sequences from H1N1, PD09, H3N2, H5N1 and H7N9 were downloaded from GenBank and Global Initiative on Sharing All Influenza Data (GISAID) EpifluTM databases. The quantity in a cell indicates the total number of virus strains in which both of their targeted segment sequences are available from either five subtypes in (A) 5-S set or from three subtypes (H1N1, PD09 and H3N2) in (B) 3-S set. (DOCX) [file pcbi.1004663.s003.docx]

**Table S3. Number of virus strains analysed in pairing of target segments**

| **A** | **Segment** | **2** | **3** | **5** | **7** | **8** |
| --- | --- | --- | --- | --- | --- | --- |
|  | **1** | 10,478 | 10,419 | 10,457 | 10,546 | 10,549 |
|  | **2** |  | 10,400 | 10,425 | 10,442 | 10,497 |
|  | **3** |  |  | 10,567 | 10,455 | 10,420 |
|  | **5** |  |  |  | 10,612 | 10,549 |
|  | **7** |  |  |  |  | 10,816 |

| **B** | **Segment** | **2** | **3** | **5** | **7** | **8** |
| --- | --- | --- | --- | --- | --- | --- |
|  | **1** | 10,916 | 10,913 | 11,391 | 16,079 | 11,432 |
|  | **2** |  | 10,930 | 11,421 | 16,181 | 11,482 |
|  | **3** |  |  | 11,217 | 16,106 | 11,497 |
|  | **5** |  |  |  | 16,465 | 11,884 |
|  | **7** |  |  |  |  | 16,394 |

Available human infecting virus strains and their segment sequences from H1N1, PD09, H3N2, H5N1 and H7N9 were downloaded from GenBank and Global Initiative on Sharing All Influenza Data (GISAID) Epiflu^TM^ databases. The quantity in a cell indicates the total number of virus strains in which both of their targeted segment sequences are available from either five subtypes in **(A)** 5-S set or from three subtypes (H1N1, PD09 and H3N2) in **(B)** 3-S set.
