# Supplementary material for: Discovery of Influenza A Virus Sequence Pairs and Their Combinations for Simultaneous Heterosubtypic Targeting that Hedge against Antiviral Resistance
Source: PLoS Comput Biol. 2016 Jan 15;12(1):e1004663. doi: 10.1371/journal.pcbi.1004663 (PMC4714944; doi:10.1371/journal.pcbi.1004663)
Supplement: S4 Table — (A) Number of target sequences in 5-S and 3-S sets that were found (up to one mismatch) in the transcriptomes and genomes of human, pig and chicken hosts. The % column tabulates the percentage of hit target sequences in the total target sequence in each set. (B) Number of human genes (with and without expression data) that were mapped from the accessions for which the viral target sequences was found. (DOCX) [file pcbi.1004663.s004.docx]

**Table S4. Hit target sequences in the transcriptomes and genomes from human, pig and chicken hosts**

| A | Blast-hits | *5-S* | | *3-S* | |
| --- | --- | --- | --- | --- | --- |
|  | ***Transcriptome*** | **Target sequences** | **%** | **Target sequences** | **%** |
|  | Human | 56 | 4.7% | 165 | 3.0% |
|  | Pig | 55 | 4.6% | 130 | 2.4% |
|  | Chicken | 43 | 3.6% | 134 | 2.4% |
|  | ***Genome*** | **Target sequences** | **%** | **Target sequences** | **%** |
|  | Human | 251 | 21.2% | 688 | 12.5% |
|  | Pig | 199 | 16.8% | 612 | 11.1% |
|  | Chicken | 260 | 22.0% | 703 | 12.7% |

| B | Blast-hits in the human transcriptome | *5-S* | *3-S* |
| --- | --- | --- | --- |
|  | *Accessions mapped* | 178 | 516 |
|  | *Genes mapped* | 36 | 133 |
|  | *Genes with expression data* | 27 (45)* | 89 (122)* |
|  | *Genes with no expression data* | 9 (11)* | 44 (43)* |

** Number of target sequences with blast-hits to the human transcriptome*

**(A)** Number of target sequences in 5-S and 3-S sets that were found (up to one mismatch) in the transcriptomes and genomes of human, pig and chicken hosts. The % column tabulates the percentage of hit target sequences in the total target sequence in each set. **(B)** Number of human genes (with and without expression data) that were mapped from the accessions for which the viral target sequences was found.
