# Supplementary material for: Discovery of Influenza A Virus Sequence Pairs and Their Combinations for Simultaneous Heterosubtypic Targeting that Hedge against Antiviral Resistance
Source: PLoS Comput Biol. 2016 Jan 15;12(1):e1004663. doi: 10.1371/journal.pcbi.1004663 (PMC4714944; doi:10.1371/journal.pcbi.1004663)
Supplement: S5 Table — 11.5% of the total unique internal segment sequences that do not belong to complete genomes were removed. Counts from the all-genome analysis are given in parentheses for comparison. (A) Target sequences. No target sequence can achieve 100% heterosubtypic coverage in the all-genome analysis. * Except for 15 new target sequences in segment 7 in the 3-S set, the target sequences in both 5-S and 3-S sets are identical in both all- and complete-genome analyses. (B) Effective Duals. All effective Duals from the all-genome analysis are complete subset of those from the complete-genome analysis. (C) Effective Doubles. All effective Doubles from the all-genome analysis are complete subset of those from the complete-genome analysis. (D) Size distribution of all 6-vertices segment partner graphs formed by a target sequence (whose NSP = 5) from each of the six internal segments (Figs 3C and S9B). * Complete graphs of size 15. (DOCX) [file pcbi.1004663.s005.docx]

**Table S5. Complete heterosubtypic coverage and resistance hedging when only viral strains with complete-genome were analysed**

| A | Target sequences | *5-S* | *3-S* |
| --- | --- | --- | --- |
|  | **Segment** | **Counts** | **Counts** |
|  | **1** | 156 (156) | 2,722 (2,722) |
|  | **2** | 243 (243) | 279 (279) |
|  | **3** | 49 (49) | 493 (493) |
|  | **5** | 107 (107) | 281 (281) |
|  | **7** | 594 (594) | 1,711* (1,696) |
|  | **8** | 34 (34) | 52 (52) |
|  | **Total** | **1,183 (1,183)** | **5,538 (5,523)** |

| B | Effective *Duals* | *5-S* | *3-S* |
| --- | --- | --- | --- |
|  | **Segment** | **Counts** | **Counts** |
|  | **1** | 952 (943) | 16,162 (15,363) |
|  | **2** | 36 (36) | 42 (42) |
|  | **3** | 102 (96) | 6,996 (6,971) |
|  | **5** | – | 4,194 (4,167) |
|  | **7** | 2,696 (587) | 11,510 (2,578) |
|  | **8** | – | 3 (3) |
|  | **Total** | **3,786 (1,662)** | **38,907 (29,124)** |

**C**

|  | Effective *Doubles* | *5-S* | | | | | *3-S* | | | | |
| --- | --- | --- | --- | --- | --- | --- | --- | --- | --- | --- | --- |
|  | **Segment** | **2** | **3** | **5** | **7** | **8** | **2** | **3** | **5** | **7** | **8** |
|  | **1** | 510  (510) | 729  (645) | 6.821  (6,821) | 5,853  (5,853) | 117  (117) | 7,970  (7,598) | 103,857  (102,840) | 36,287  (36,287) | 65,369  (63,335) | 5,601  (4,831) |
|  | **2** |  | 414  (414) | 2,280  (2,280) | 2,634  (2,634) | 72  (72) |  | 4,233  (4,107) | 4,482  (4,482) | 3,891  (3,828) | 162  (162) |
|  | **3** |  |  | 3,687  (3,687) | 945  (879) | 130  (108) |  |  | 28,971  (28,971) | 12,724  (11,710) | 2,255  (2,233) |
|  | **5** |  |  |  | 3,033  (2,946) | 468  (468) |  |  |  | 7,833  (6,807) | 1,404  (1,404) |
|  | **7** |  |  |  |  | 1,029  (1,029) |  |  |  |  | 1,762  (1,756) |
|  | **Total** | **28,722 (28,463)** | | | | | **286,801 (280,351)** | | | | |

| D | Graphs size distribution | *5-S* | *3-S* |
| --- | --- | --- | --- |
|  | **Graph size** | **Counts** | **Counts** |
|  | **2** | – | 539,518,482 (539,518,482) |
|  | **3** | – | 1,354,611,060 (1,359,713,196) |
|  | **4** | – | 2,223,713,007 (2,268,239,631) |
|  | **5** | – | 4,076,585,568 (4,150,228,191) |
|  | **6** | – | 5,977,488,465 (5,972,694,501) |
|  | **7** | – | 7,239,244,167 (7,372,942,287) |
|  | **8** | – | 6,031,623,564 (6,130,754,955) |
|  | **9** | – | 5,731,529,589 (5,769,792,072) |
|  | **10** | 126,360 (126,360) | 4,416,269,760 (4,280,931,897) |
|  | **11** | 909,792 (909,792) | 3,024,596,268 (2,916,639,192) |
|  | **12** | 1,794,312 (1,794,312) | 1,701,899,712 (1,610,712,312) |
|  | **13** | 4,245,696 (4,245,696) | 621,533,466 (575,618,256) |
|  | **14** | 3,790,800 (3,790,800) | 97,891,848 (89,329,752) |
|  | **15 *** | 808,704 (808,704) | 4,554,144 (3,944,376) |
|  | **Total** | **11,675,664 (11,675,664)** | **43,041,059,100 (43,041,059,100)** |

11.5% of the total unique internal segment sequences that do not belong to complete genomes were removed. Counts from the all-genome analysis are given in parentheses for comparison. **(A)** Target sequences. No target sequence can achieve 100% heterosubtypic coverage in the all-genome analysis. * Except for 15 new target sequences in segment 7 in the 3-S set, the target sequences in both 5-S and 3-S sets are identical in both all- and complete-genome analyses. **(B)** Effective *Duals*. All effective *Duals* from the all-genome analysis are complete subset of those from the complete-genome analysis. **(C)** Effective *Doubles*. All effective *Doubles* from the all-genome analysis are complete subset of those from the complete-genome analysis. **(D)** Size distribution of all 6-vertices segment partner graphs formed by a target sequence (whose *NSP = 5*) from each of the six internal segments (Fig. 3C and Fig. S9B). * Complete graphs of size 15.
