# Supplementary material for: Discovery of Influenza A Virus Sequence Pairs and Their Combinations for Simultaneous Heterosubtypic Targeting that Hedge against Antiviral Resistance
Source: PLoS Comput Biol. 2016 Jan 15;12(1):e1004663. doi: 10.1371/journal.pcbi.1004663 (PMC4714944; doi:10.1371/journal.pcbi.1004663)
Supplement: S6 Table — 11% of the total unique internal segment sequences that are non-full-length were removed. Counts from the all-length segment sequence analysis are given in parentheses for comparison. (A) Target sequences. No target sequence can achieve 100% heterosubtypic coverage in the all-genome analysis. No single target sequence can achieve 100% heterosubtypic coverage in the full-length analysis. * Except for one target sequence in segment 7 in the 3-S set, the target sequences in both 5-S and 3-S sets are identical in both full- and all-length analyses. (B) Effective Duals. * Except for five effective Duals in segment 7 in the 3-S set, all effective Duals from the all-length analysis are complete subset of those from the full-length analysis. ** 21 effective Duals in segment 5 were obtained in the full-length analysis whereas there was none in the all-length analysis. (C) Effective Doubles. * Except for six S1-S7, 45 S5-S7, and three S7-S8 effective Doubles in the 3-S set, all effective Doubles from the all-length analysis are complete subset of those from the full-length analysis. (D) Size distribution of all 6-vertices segment partner graphs formed by a target sequence (whose NSP = 5) from each of the six internal segments (Figs 3C and S9B). *Complete graphs of size 15. (DOCX) [file pcbi.1004663.s006.docx]

**Table S6. Complete heterosubtypic coverage and resistance hedging when only full-length viral segment sequences were analysed**

| A | Target sequences | *5-S* | *3-S* |
| --- | --- | --- | --- |
|  | **Segment** | **Counts** | **Counts** |
|  | **1** | 156 (156) | 2,722 (2,722) |
|  | **2** | 243 (243) | 279 (279) |
|  | **3** | 49 (49) | 493 (493) |
|  | **5** | 107 (107) | 281 (281) |
|  | **7** | 594 (594) | 1,695* (1,696) |
|  | **8** | 34 (34) | 52 (52) |
|  | **Total** | **1,183 (1,183)** | **5,522 (5,523)** |

| B | Effective *Duals* | *5-S* | *3-S* |
| --- | --- | --- | --- |
|  | **Segment** | **Counts** | **Counts** |
|  | **1** | 957 (943) | 15,363 (15,363) |
|  | **2** | 36 (36) | 42 (42) |
|  | **3** | 156 (96) | 28,676 (6,971) |
|  | **5** | 21** (0) | 4,939* (4,167) |
|  | **7** | 1,230 (587) | 4,249 (2,578) |
|  | **8** | – | 3 (3) |
|  | **Total** | **2,400 (1,662)** | **53,272 (29,124)** |

**C**

|  | Effective *Doubles* | *5-S* | | | | | *3-S* | | | | |
| --- | --- | --- | --- | --- | --- | --- | --- | --- | --- | --- | --- |
|  | **Segment** | **2** | **3** | **5** | **7** | **8** | **2** | **3** | **5** | **7** | **8** |
|  | **1** | 528  (510) | 696  (645) | 6.827  (6,821) | 5,853  (5,853) | 285  (117) | 7,598  (7,598) | 103,917  (102,840) | 38,471  (36,287) | 75,816*  (63,335) | 6,137  (4,831) |
|  | **2** |  | 414  (414) | 2,280  (2,280) | 3,114  (2,634) | 162  (72) |  | 6,402  (4,107) | 4,482  (4,482) | 4,524  (3,828) | 312  (162) |
|  | **3** |  |  | 3,687  (3,687) | 969  (879) | 276  (108) |  |  | 35,761  (28,971) | 15,235  (11,710) | 3,469  (2,233) |
|  | **5** |  |  |  | 8,322  (2,946) | 468  (468) |  |  |  | 14,538*  (6,807) | 1,449  (1,404) |
|  | **7** |  |  |  |  | 1,749  (1,029) |  |  |  |  | 3,631*  (1,756) |
|  | **Total** | **35,630 (28,463)** | | | | | **321,742 (280,351)** | | | | |

| D | Graphs size distribution | *5-S* | *3-S* |
| --- | --- | --- | --- |
|  | **Graph size** | **Counts** | **Counts** |
|  | **1** | – | 497,386,251 (0) |
|  | **2** | – | 7,356,828,159 (539,518,482) |
|  | **3** | – | 13,311,685,395 (1,359,713,196) |
|  | **4** | – | 25,446,077,829 (2,268,239,631) |
|  | **5** | – | 41,395,204,197 (4,150,228,191) |
|  | **6** | 31,050 (0) | 58,605,029,064 (5,972,694,501) |
|  | **7** | 403,380 (0) | 65,331,607,806 (7,372,942,287) |
|  | **8** | 1,275,156 (0) | 57,289,184,010 (6,130,754,955) |
|  | **9** | 2,995,488 (0) | 41,714,899,560 (5,769,792,072) |
|  | **10** | 9,397,134 (126,360) | 28,921,666,005 (4,280,931,897) |
|  | **11** | 18,730,980 (909,792) | 16,146,455,949 (2,916,639,192) |
|  | **12** | 24,155,280 (1,794,312) | 7,458,580,143 (1,610,712,312) |
|  | **13** | 15,426,612 (4,245,696) | 2,014,509,366 (575,618,256) |
|  | **14** | 8,505,864 (3,790,800) | 302,888,754 (89,329,752) |
|  | **15 *** | 808,704 (808,704) | 7,056,072 (3,944,376) |
|  | **Total** | **81,729,648 (11,675,664)** | **365,799,058,560 (43,041,059,100)** |

11% of the total unique internal segment sequences that are non-full-length were removed. Counts from the all-length segment sequence analysis are given in parentheses for comparison. **(A)** Target sequences. No target sequence can achieve 100% heterosubtypic coverage in the all-genome analysis. No single target sequence can achieve 100% heterosubtypic coverage in the full-length analysis. * Except for one target sequence in segment 7 in the 3-S set, the target sequences in both 5-S and 3-S sets are identical in both full- and all-length analyses. **(B)** Effective *Duals*. * Except for five effective *Duals* in segment 7 in the 3-S set, all effective *Duals* from the all-length analysis are complete subset of those from the full-length analysis. ** 21 effective *Duals* in segment 5 were obtained in the full-length analysis whereas there was none in the all-length analysis. **(C)** Effective *Doubles*. * Except for six S1-S7, 45 S5-S7, and three S7-S8 effective *Doubles* in the 3-S set, all effective *Doubles* from the all-length analysis are complete subset of those from the full-length analysis. **(D)** Size distribution of all 6-vertices segment partner graphs formed by a target sequence (whose *NSP = 5*) from each of the six internal segments (Fig. 3C and Fig. S9B). *Complete graphs of size 15.
