# Supplementary material for: Discovery of Influenza A Virus Sequence Pairs and Their Combinations for Simultaneous Heterosubtypic Targeting that Hedge against Antiviral Resistance
Source: PLoS Comput Biol. 2016 Jan 15;12(1):e1004663. doi: 10.1371/journal.pcbi.1004663 (PMC4714944; doi:10.1371/journal.pcbi.1004663)
Supplement: S1 Text — (DOCX) [file pcbi.1004663.s007.docx]

**Text S1. Description of data resources**

A total of 39 resource files are provided in this manuscript, as tabulated below. The description and data formats are given in the following.

| # | Filename | File size (MB) |
| --- | --- | --- |
| 1 | *uid2Zsequence_consolidated.txt* | 3  (compressed into Data-source.7z) |
| 2 | *H1N1-strainnames2-date-and-uids.txt* |  |
| 3 | *PD09-strainnames2-date-and-uids.txt* |  |
| 4 | *H3N2-strainnames2-date-and-uids.txt* |  |
| 5 | *H5N1-strainnames2-date-and-uids.txt* |  |
| 6 | *H7N9-strainnames2-date-and-uids.txt* |  |
| 7 | *aH1N1-strainnames2-date-and-uids.txt* |  |
| 8 | *aH3N2-strainnames2-date-and-uids.txt* |  |
| 9 | *aH5N1-strainnames2-date-and-uids.txt* |  |
| 10 | *aH7N9-strainnames2-date-and-uids.txt* |  |
| 11 | *H00N00-strainnames2-date-and-uids.txt* |  |
| 12 | *zoonotic-strainnames2-date-and-uids.txt* |  |
| 13 | *exotic-strainnames2-date-and-uids.txt* |  |
| 14 | *strainname-subtype-segment-accessionurl.txt* |  |
| 15 | *Full-segment-consensus.txt* | 0.06 |
| 16 | *5S-targetSequences.txt* | 14  (compressed into 5-S_Results.7z) |
| 17 | *5S-EffectiveDuals.txt* |  |
| 18 | *5S-EffectiveDoubles.txt* |  |
| 19 | *5S-targetSequences_noRNAHits.txt* |  |
| 20 | *5S-EffectiveDuals_noRNAHits.txt* |  |
| 21 | *5S-EffectiveDoubles_noRNAHits.txt* |  |
| 22 | *5S-targetSequences_noDNAnRNAHits.txt* |  |
| 23 | *5S-EffectiveDuals_noDNAnRNAHits.txt* |  |
| 24 | *5S-EffectiveDoubles_noDNAnRNAHits.txt* |  |
| 25 | *5S-Hits.txt* |  |
| 26 | *5S-Hits_TissueExpressions.txt* |  |
| 27 | *5S-bindingAccessibilities.txt* |  |
| 28 | *3S-targetSequences.txt* | 72  (compressed into 3-S_Results.7z) |
| 29 | *3S-EffectiveDuals.txt* |  |
| 30 | *3S-EffectiveDoubles.txt* |  |
| 31 | *3S-targetSequences_noRNAHits.txt* |  |
| 32 | *3S-EffectiveDuals_noRNAHits.txt* |  |
| 33 | *3S-EffectiveDoubles_noRNAHits.txt* |  |
| 34 | *3S-targetSequences_noDNAnRNAHits.txt* |  |
| 35 | *3S-EffectiveDuals_noDNAnRNAHits.txt* |  |
| 36 | *3S-EffectiveDoubles_noDNAnRNAHits.txt* |  |
| 37 | *3S-Hits.txt* |  |
| 38 | *3S-Hits_TissueExpressions.txt* |  |
| 39 | *3S-bindingAccessibilities.txt* |  |

Note: the files can be downloaded at <http://mendel.bii.a-star.edu.sg/SEQUENCES/HEDGING_DRUG_RESISTANCE/SUPPLEMENTARY/>

*Segment sequences and subtype strains*

Every unique segment sequence (including its UTRs, if any) is given a unique segment sequence-ID of the format {*subtype*}-{*segment*}-{unique *running number*} for example, *H1N1-1-20*. All the unique segment sequences from human and animal strains analysed in this study were listed in *uid2Zsequence_consolidated.txt*. The strains and their corresponding segment sequence-IDs for subtypes H1N1, PD09, H3N2, H5N1, H7N9, aH1N1, aH3N2, aH5N1, aH7N9, and groups *H00N00*, *zoonotic* and *exotic* are given in respectively, *H1N1-strainnames2-date-and-uids.txt*, *PD09-strainnames2-date-and-uids.txt*, *H3N2-strainnames2-date-and-uids.txt*, *H5N1-strainnames2-date-and-uids.txt*, *H7N9-strainnames2-date-and-uids.txt*, *aH1N1-strainnames2-date-and-uids.txt*, *aH3N2-strainnames2-date-and-uids.txt*, *aH5N1-strainnames2-date-and-uids.txt*, *aH7N9-strainnames2-date-and-uids.txt*, *H00N00-strainnames2-date-and-uids.txt*, *zoonotic-strainnames2-date-and-uids.txt* and *exotic-strainnames2-date-and-uids.txt*. Finally, the Accession URLs of each unique segment sequence analysed are listed in *strainname-subtype-segment-accessionurl.txt*. Below summarizes the tab delimited columns in each file.

*(#1) uid2Zsequence_consolidated.txt*

| Column | Description |
| --- | --- |
| 1 | Segment sequence-ID |
| 2 | Segment sequence (for non-full-length sequence, a “Z” is used to indicate each unreported base) |
| 3 | Boundary of coding region in the sequence |

Note: due to licensing terms, sequences extracted from GISAID cannot be given in the file. Readers are required to register for an account at <http://platform.gisaid.org/> in order to download the sequences.

*(#2) H1N1-strainnames2-date-and-uids.txt*

*(#3) PD09-strainnames2-date-and-uids.txt*

*(#4) H3N2-strainnames2-date-and-uids.txt*

*(#5) H5N1-strainnames2-date-and-uids.txt*

*(#6) H7N9-strainnames2-date-and-uids.txt*

*(#7) aH1N1-strainnames2-date-and-uids.txt*

*(#8) aH3N2-strainnames2-date-and-uids.txt*

*(#9) aH5N1-strainnames2-date-and-uids.txt*

*(#10) aH7N9-strainnames2-date-and-uids.txt*

*(#11) H00N00-strainnames2-date-and-uids.txt*

*(#12) zoonotic-strainnames2-date-and-uids.txt*

*(#13) exotic-strainnames2-date-and-uids.txt*

| Column | Description |
| --- | --- |
| 1 | Full strain name |
| 2 | Curation date (*yyyymmdd*) |
| 3 | Unique sequence-ID (segment 1) |
| 4 | Unique sequence-ID (segment 2) |
| 5 | Unique sequence-ID (segment 3) |
| 6 | Unique sequence-ID (segment 4) (applicable to H1N1, PD09, H3N2, H5N1 and H7N9 only) |
| 7 | Unique sequence-ID (segment 5) |
| 8 | Unique sequence-ID (segment 6) (applicable to H1N1, PD09, H3N2, H5N1 and H7N9 only) |
| 9 | Unique sequence-ID (segment 7) |
| 10 | Unique sequence-ID (segment 8) |

*(#14) strainname-subtype-segment-accessionurl.txt*

| Column | Description |
| --- | --- |
| 1 | Full strain name |
| 2 | Subtype name |
| 3 | Segment |
| 4 | Accession URL |
| 5 | Segment sequence-ID |

*Full segment consensus sequences*

The full segment consensus sequence of segments 1, 2, 3, 5, 7, and 8 for H1N1, PD09, H3N2, H5N1 and H7N9 subtypes are given in *(#15) Full-segment-consensus.txt*.

*Target sequences*

For each target sequence, it is assigned a unique target ID of the format {*segment*}.{*target sequence starting position*}.{*target length*} for example, *S1.50.15*; the target sequence position is with reference to the respective full segment consensus sequence. The full list of target sequences identified in 5-S and 3-S sets and each of their corresponding properties are given respectively in *(#16)* *5S-targetSequences.txt* and *(#28)* *3S-targetSequences.txt*, in *(#19)* *5S-targetSequences_noRNAHits.txt* and *(#31)* *3S-targetSequences_noRNAHits.txt* in the absence of hit target sequences to the human transcriptome, and in *(#22)* *5S-targetSequences_noDNAnRNAHits.txt* and *(#34)* *3S-targetSequences_noDNAnRNAHits.txt* in the absence of hit target sequences to the human genome and transcriptome. Data fields in the files are described as follows.

| Column | Description |
| --- | --- |
| 1 | Target ID |
| 2 | Coverage % against H1N1 |
| 3 | Coverage % against PD09 |
| 4 | Coverage % against H3N2 |
| 5 | Coverage % against H5N1 (applicable to 5-S set only) |
| 6 | Coverage % against H7N9 (applicable to 5-S set only) |
| 7 | Unmatched H1N1 segment sequence-ID: segment sequences (if any, delimited by “,”) in which a complete match of the current target sequence is not found |
| 8 | Unmatched PD09 segment sequence-ID |
| 9 | Unmatched H3N2 segment sequence-ID |
| 10 | Unmatched H5N1 segment sequence-ID (applicable to 5-S set only) |
| 11 | Unmatched H7N9 segment sequence-ID (applicable to 5-S set only) |

*Effective Duals*

Combinations of single target sequences in 5-S and 3-S sets that resulted in effective *Duals* are listed respectively in *(#17) 5S-EffectiveDuals.txt and (#29) 3S-EffectiveDuals.txt*, in *(#20)* *5S-EffectiveDuals_noRNAHits.txt* and *(#32)* *3S-EffectiveDuals_noRNAHits.txt* in the absence of hit target sequences to the human transcriptome, and in *(#23)* *5S-EffectiveDuals_noDNAnRNAHits.txt* and *(#35)* *3S-EffectiveDuals_noDNAnRNAHits.txt* in the absence of hit target sequences to the human genome and transcriptome.

*Effective Doubles*

Combinations of single target sequences in 5-S and 3-S sets that resulted in effective *Doubles* are listed respectively in *(#18)* *5S-EffectiveDoubles.txt and (#30)* *3S-EffectiveDoubles.txt*, in *(#21)* *5S-EffectiveDoubles_noRNAHits.txt* and *(#33)* *3S-EffectiveDoubles_noRNAHits.txt* in the absence of hit target sequences to the human transcriptome, and in *(#24)* *5S-EffectiveDoubles_noDNAnRNAHits.txt* and *(#36)* *3S-EffectiveDoubles_noDNAnRNAHits.txt* in the absence of hit target sequences to the human genome and transcriptome.

*Hit target sequences to the genome and transcriptomes of human, pig and chicken hosts*

Target sequences in 5-S and 3-S sets that were found (up to one mismatch) in the genomes or transcriptomes of human, pig or chicken hosts and the associated accessions are given in *(#25) 5S-Hits.txt* and *(#37) 3S-Hits.txt*, respectively. Data fields in the files are described as follows.

| Column | Description |
| --- | --- |
| 1 | Target ID |
| 2 | Human transcriptome (1:complete match; 1-m: 1-mismatch) |
| 3 | Pig transcriptome (1:complete match; 1-m: 1-mismatch) |
| 4 | Chicken transcriptome (1:complete match; 1-m: 1-mismatch) |
| 5 | Human genome (1:complete match; 1-m: 1-mismatch) |
| 6 | Pig genome (1:complete match; 1-m: 1-mismatch) |
| 7 | Chicken genome (1:complete match; 1-m: 1-mismatch) |
| 8 | Human transcriptome accession(s), delimited by “\|” |
| 9 | Pig transcriptome accession(s), delimited by “\|” |
| 10 | Chicken transcriptome accession(s), delimited by “\|” |
| 11 | Human genome accession(s), delimited by “\|” |
| 12 | Pig genome accession(s), delimited by “\|” |
| 13 | Chicken genome accession(s), delimited by “\|” |

The human transcriptome accessions were mapped to their respective genes and their expression status in 84 tissues and cell types where available, as given in *(#26) 5S-Hits_TissueExpressions.txt* and *(#38) 3S-Hits_TissueExpressions.txt*, respectively in 5-S and 3-S sets. Data fields in the files are described as follows.

| Column | Description |
| --- | --- |
| 1 | Target ID |
| 2 | Human transcriptome accession(s), delimited by “\|” |
| 3 | Entrez Gene ID |
| 4 | Gene Name |
| 5 | Microarray Probe ID |
| 6 | Expression in |
| 7 | Expression in 721_B_lymphoblasts |
| 8 | Expression in Adipocyte |
| 9 | Expression in AdrenalCortex |
| 10 | Expression in Adrenalgland |
| 11 | Expression in Amygdala |
| 12 | Expression in Appendix |
| 13 | Expression in AtrioventricularNode |
| 14 | Expression in BDCA4+_DentriticCells |
| 15 | Expression in Bonemarrow |
| 16 | Expression in BronchialEpithelialCells |
| 17 | Expression in CD105+_Endothelial |
| 18 | Expression in CD14+_Monocytes |
| 19 | Expression in CD19+_BCells(neg._sel.) |
| 20 | Expression in CD33+_Myeloid |
| 21 | Expression in CD34+ |
| 22 | Expression in CD4+_Tcells |
| 23 | Expression in CD56+_NKCells |
| 24 | Expression in CD71+_EarlyErythroid |
| 25 | Expression in CD8+_Tcells |
| 26 | Expression in CardiacMyocytes |
| 27 | Expression in Caudatenucleus |
| 28 | Expression in Cerebellum |
| 29 | Expression in CerebellumPeduncles |
| 30 | Expression in CiliaryGanglion |
| 31 | Expression in CingulateCortex |
| 32 | Expression in Colorectaladenocarcinoma |
| 33 | Expression in DorsalRootGanglion |
| 34 | Expression in FetalThyroid |
| 35 | Expression in Fetalbrain |
| 36 | Expression in Fetalliver |
| 37 | Expression in Fetallung |
| 38 | Expression in GlobusPallidus |
| 39 | Expression in Heart |
| 40 | Expression in Hypothalamus |
| 41 | Expression in Kidney |
| 42 | Expression in Leukemia_chronicMyelogenousK-562 |
| 43 | Expression in Leukemia_promyelocytic-HL-60 |
| 44 | Expression in Leukemialymphoblastic(MOLT-4) |
| 45 | Expression in Liver |
| 46 | Expression in Lung |
| 47 | Expression in Lymphnode |
| 48 | Expression in Lymphoma_burkitts(Daudi) |
| 49 | Expression in Lymphoma_burkitts(Raji) |
| 50 | Expression in MedullaOblongata |
| 51 | Expression in OccipitalLobe |
| 52 | Expression in OlfactoryBulb |
| 53 | Expression in Ovary |
| 54 | Expression in Pancreas |
| 55 | Expression in PancreaticIslet |
| 56 | Expression in ParietalLobe |
| 57 | Expression in Pituitary |
| 58 | Expression in Placenta |
| 59 | Expression in Pons |
| 60 | Expression in PrefrontalCortex |
| 61 | Expression in Prostate |
| 62 | Expression in Salivarygland |
| 63 | Expression in SkeletalMuscle |
| 64 | Expression in Skin |
| 65 | Expression in SmoothMuscle |
| 66 | Expression in Spinalcord |
| 67 | Expression in SubthalamicNucleus |
| 68 | Expression in SuperiorCervicalGanglion |
| 69 | Expression in TemporalLobe |
| 70 | Expression in Testis |
| 71 | Expression in TestisGermCell |
| 72 | Expression in TestisIntersitial |
| 73 | Expression in TestisLeydigCell |
| 74 | Expression in TestisSeminiferousTubule |
| 75 | Expression in Thalamus |
| 76 | Expression in Thymus |
| 77 | Expression in Thyroid |
| 78 | Expression in Tongue |
| 79 | Expression in Tonsil |
| 80 | Expression in Trachea |
| 81 | Expression in TrigeminalGanglion |
| 82 | Expression in Uterus |
| 83 | Expression in UterusCorpus |
| 84 | Expression in WholeBlood |
| 85 | Expression in Wholebrain |
| 86 | Expression in colon |
| 87 | Expression in pineal_day |
| 88 | Expression in pineal_night |
| 89 | Expression in retina |
| 90 | Expression in small_intestine |

*Target sequences binding accessibilities*

The secondary structure of each segment sequence was predicted using mfold (Zuker M. Mfold web server for nucleic acid folding and hybridization prediction. Nucleic Acids Res 2003 31:3406) for which all optimal and sub-optimal structures were used to compute *AVG* and *L3* scores. The *AVG* score of a target sequence in a segment sequence estimates the average binding accessibility of every target nucleotide in a segment sequence; a high *AVG* (*0 ≤ AVG ≤ 1*) suggests the target site is accessibility for antisense oligonucleotide binding. It is computed as,

$$AVG=\frac{\sum_{i=1}^{n} Number of structures i^{th} target nucleotide is unbound}{Total structures*n}, n=target sequence length$$

On the other hand, the *L3* score of a target sequence in a segment sequence estimates the average occurrence of “*engaged-nucleotides*” within the target site in a segment sequence; a target nucleotide is termed as *engaged* when it is bounded (thus, inaccessible for antisense oligonucleotide binding) in all the secondary structures predicted; a low *L3* (*0 ≤ L3 ≤ 1*) suggests the target site is less likely to have *engaged-nucleotides* (Wee KB, Pramono ZA, Wang JL, MacDorman KF, Lai PS, Yee WC. Dynamics of *co-transcriptional* pre-mRNA folding influences the induction of dystrophin exon skipping by antisense oligonucleotides. PLoS One 2008 3:e1844). It is computed as,

$$L3=\frac{\sum_{i=1}^{n} Engaged nucleotides}{n}, n=target sequence length$$

Both the *AVG* and *L3* scores were computed in all segment sequences which the target sequence is found. The scores are given respectively in *(#27)* *5S-bindingAccessibilities.txt* and *(#39)* *3S-bindingAccessibilities.txt* for target sequences in 5-S and 3-S sets. Data fields in the files are described as follows.

| Col | Description |
| --- | --- |
| 1 | Target ID |
| 2 | Binding accessibility score (AVG) among H1N1 strains delimited by “,” |
| 3 | Binding accessibility score (AVG) among PD09 strains delimited by “,” |
| 4 | Binding accessibility score (AVG) among H3N2 strains delimited by “,” |
| 5 | Binding accessibility score (AVG) among H5N1 strains delimited by “,” (applicable to the 5-S set only) |
| 6 | Binding accessibility score (AVG) among H7N9 strains delimited by “,” (applicable to the 5-S set only) |
| 7 | “*Engaged-nucleotide*” score (L3) among H1N1 strains delimited by “,” |
| 8 | “*Engaged-nucleotide*” score (L3) among PD09 strains delimited by “,” |
| 9 | “*Engaged-nucleotide*” score (L3) among H3N2 strains delimited by “,” |
| 10 | “*Engaged-nucleotide*” score (L3) among H5N1 strains delimited by “,” (applicable to the 5-S set only) |
| 11 | “*Engaged-nucleotide*” score (L3) among H7N9 strains delimited by “,” (applicable to the 5-S set only) |
