# Supplementary material for: Discovery of Influenza A Virus Sequence Pairs and Their Combinations for Simultaneous Heterosubtypic Targeting that Hedge against Antiviral Resistance
Source: PLoS Comput Biol. 2016 Jan 15;12(1):e1004663. doi: 10.1371/journal.pcbi.1004663 (PMC4714944; doi:10.1371/journal.pcbi.1004663)
Supplement: S2 Text — (DOCX) [file pcbi.1004663.s008.docx]

**Text S2. Monte Carlo simulations for estimating mutation counts and time to resistance**

Monte Carlo simulations were performed to estimate the mutation counts and time elapsed for a set of target sequences to become resistant. A set containing *n* target sequences is considered resistant when *n – 1* target sequences are resistant; a target sequence becomes resistant when it acquires one mutation in the 1-hit study (Fig. 5) or three mutations in the 3-hits study (Fig. S14). In the model, random single nucleotide substitution events are simulated in each of the eight viral segments. The model assumptions are:

1. The probability of each nucleotide in a segment to acquire a substitution mutation is modelled by a uniform distribution. Although synonymous and non-synonymous substitutions were observed to have different mutation frequencies [R1-R2], the mutation rates are apparent as substitutions that lead to virus unviability will not be observed. The model does not exclude substitutions that are not viable for the virus as it is not possible to accurately predict the differential impact of non-synonymous substitutions on the virus viability and fitness. As a result, the simulation results could underestimate the mutation counts and elapsed time required for a set of target sequences to become resistant since it includes all mutation events.
2. The nucleotide substitution probability is not affected by the targeting of RNA therapeutics.
3. Substitution events among nucleotides in a same segment and between segments are independent.

Segment mutation rates:

The mutation rates (substitutions per year) of the eight segments are tabulated below which are derived from previous report [R3].

| Segment | Substitutions/year | Pr(Segment) |
| --- | --- | --- |
| 1 | 3.876 | 0.15833398 |
| 2 | 3.6384 | 0.148628058 |
| 3 | 4.0869 | 0.166949211 |
| 4 | 4.4997 | 0.183812025 |
| 5 | 2.994 | 0.122304421 |
| 6 | 3.3135 | 0.135355945 |
| 7 | 0.982 | 0.040114543 |
| 8 | 1.0894 | 0.044501816 |
| Total | **24.4799** | 1 |

References for Text S2:

1. Bhatt S, Holmes EC, Pybus OG. The Genomic Rate of Molecular Adaptation of the Human Inﬂuenza A Virus. Mol Biol Evol 2011 28:2443-2451.
2. Lu L, Lycett SJ, Brown AJL. Reassortment patterns of avian influenza virus internal segments among different subtypes. BMC Evol Biol 2014 14:16.
3. Worobey M, Han GZ, Rambaut A. A synchronized global sweep of the internal genes of modern avian influenza virus. Nature 2014 508:254-257.
