# Supplementary material for: Discovery of Influenza A Virus Sequence Pairs and Their Combinations for Simultaneous Heterosubtypic Targeting that Hedge against Antiviral Resistance
Source: PLoS Comput Biol. 2016 Jan 15;12(1):e1004663. doi: 10.1371/journal.pcbi.1004663 (PMC4714944; doi:10.1371/journal.pcbi.1004663)
Supplement: S3 Text — (DOCX) [file pcbi.1004663.s009.docx]

**Text S3. Validated target sequences**

78 validated target sequences (15 nucleotides or longer) were obtained from the literature [R4-R15] for which 47 of them (60%) were found in the segment consensus of at least one subtype (indicated in columns 3 to 7). Nine of them were found in the 3-S set of target sequences whereas five were found in the 5-S set; the corresponding target sequence IDs are given in the first column. Notably, they form effective *Duals*, effective *Doubles* and target sequence graphs when they are combined. For validated target sequences in the 3-S set, they form three effective *Duals* in segment 1, nine effective *Doubles* (between segments 1 and 2, segments 2 and 7, and segments 1 and 7), and a target sequence graph of size 12 with hedge-factor equals to two. In the 5-S set case, they form two effective *Duals* in segment 1, five effective *Doubles* (between segments 1 and 2, segments 2 and 7, and segments 1 and 7), and a target sequence graph of size 7 with hedge-factor equals to two. The IDs of the validated target sequences that form effective *Duals* and effective *Doubles* are listed below while the target sequence graphs are depicted below.

| *Segment 1 (PB2)* | | | | | | | |
| --- | --- | --- | --- | --- | --- | --- | --- |
| ID | **Target sequence** | **H1N1** | **PD09** | **H3N2** | **H5N1** | **H7N9** | **Ref.** |
| – | GGAGACGTGGTGTTGGTAA |  |  | *X* | *X* |  | R4 |
| S1.2215.19 ^3-S, 5-S^ | CGGGACTCTAGCATACTTA | *X* | *X* | *X* | *X* | *X* | R4 |
| – | AAGAATAAAAGAACT |  |  | *X* |  | *X* | R6 |
| S1.2222.15 ^3-S, 5-S^ | CTAGCATACTTACTG | *X* | *X* | *X* | *X* | *X* | R6 |
| S1.2237.15 ^3-S^ | ACAGCCAGACAGCGA | *X* | *X* | *X* | *X* |  | R6 |
| S1.2252.15 ^3-S^ | CCAAAAGAATTCGGA | *X* | *X* | *X* | *X* |  | R6 |
| S1.2258.15 ^3-S^ | GAATTCGGATGGCCA | *X* | *X* | *X* | *X* |  | R6 |
| S1.2252.21 ^3-S^ | CCAAAAGAATTCGGATGGCCA | *X* | *X* | *X* | *X* |  | R6 |
| S1.2242.15 ^3-S, 5-S^ | CAGACAGCGACCAAA | *X* | *X* | *X* | *X* | *X* | R7 |
| *Segment 2 (PB1)* | | | | | | | |
| ID | **Target sequence** | **H1N1** | **PD09** | **H3N2** | **H5N1** | **H7N9** | **Ref.** |
| S2.2235.19 ^3-S, 5-S^ | GATCTGTTCCACCATTGAA | *X* | *X* | *X* | *X* | *X* | R4 |
| – | GGAATGAGAAGAAGGCTAA |  |  |  | *X* |  | R11 |
| *Segment 3 (PA)* | | | | | | | |
| ID | **Target sequence** | **H1N1** | **PD09** | **H3N2** | **H5N1** | **H7N9** | **Ref.** |
| – | TGCTTCAATCCGATGATTG | *X* |  |  |  |  | R4 |
| – | CGGCTACATTGAGGGCAAG | *X* |  |  |  |  | R4 |
| – | GCAATTGAGGAGTGCCTGA | *X* |  | *X* | *X* |  | R4 |
| – | TGATCCCTGGGTTTTGCTT | *X* | *X* |  |  | *X* | R4 |
| – | TGCTTCTTGGTTCAACTCC | *X* |  |  |  |  | R4 |
| – | TATGAAGCAATTGAGGAGTGCCTGA | *X* |  | *X* | *X* |  | R8 |
| – | GAGAGCAGGGCAAGAATTA |  |  | *X* |  |  | R9 |
| – | GAACATGGCACCAGAGAAA |  |  | *X* |  |  | R9 |
| – | CAAAGAAGGAAGACGGAAA |  | *X* |  | *X* | *X* | R9 |
| – | CAAGTTTCAAGGCCCATGT |  |  |  | *X* |  | R11 |
| *Segment 5 (NP)* | | | | | | | |
| ID | **Target sequence** | **H1N1** | **PD09** | **H3N2** | **H5N1** | **H7N9** | **Ref.** |
| – | TAGAGAGAATGGTGCTCTC | *X* |  |  |  |  | R4 |
| – | TAAGGCGAATCTGGCGCCA |  |  | *X* |  |  | R4 |
| – | GGATCTTATTTCTTCGGAG | *X* |  | *X* | *X* | *X* | R4 |
| – | GATCCAGAACAGCATAACA |  |  |  | *X* |  | R10 |
| – | ATCCAGAACAGCATAACAA |  |  |  | *X* |  | R10 |
| – | CCAGAACAGCATAACAATA |  |  |  | *X* |  | R10 |
| – | GCATAACAATAGAGAGAAT |  |  |  | *X* |  | R10 |
| – | GCAATGGACTCCAACACTC |  |  |  | *X* |  | R11 |
| – | AGAGCAATGATGGATCAAG |  |  |  | *X* |  | R12 |
| – | TGATGGAAAGTGCCAGACC |  |  |  | *X* | *X* | R12 |
| – | AAGGAACAGATACCTGGA |  |  |  | *X* | *X* | R15 |
| – | CGGATGATAAAACGAGGG |  |  |  | *X* | *X* | R15 |
| – | AAAGAGCAATGATGGATC |  |  |  | *X* |  | R15 |
| *Segment 7 (M)* | | | | | | | |
| ID | **Target sequence** | **H1N1** | **PD09** | **H3N2** | **H5N1** | **H7N9** | **Ref.** |
| – | CCGAGGTCGAAACGTACGT | *X* | *X* |  | *X* | *X* | R4 |
| – | CAGATTGCTGACTCCCAGC |  |  | *X* |  |  | R4 |
| – | GAATATCGAAAGGAACAGC |  |  | *X* |  |  | R4 |
| – | ACAGCAGAATGCTGTGGAT | *X* |  | *X* |  | *X* | R5 |
| S7.52.15 ^3-S, 5-S^ | GGCCCCCTCAAAGCC | *X* | *X* | *X* | *X* | *X* | R7 |
| – | TGGGAGTGCAGATGCAGCG |  | *X* |  | *X* |  | R12 |
| – | CAGCAGAGTGCTGTGGATG |  | *X* |  | *X* |  | R12 |
| – | GCCGAGATCGCACAGAGAC | *X* |  |  |  | *X* | R13 |
| – | GCTTAAGAGGGAGATAACA | *X* |  |  |  |  | R13 |
| – | GGTCGAAACGTATGTTCTCTC |  |  | *X* |  |  | R14 |
| – | AATTTGCAGGCCTATCAGAAA | *X* |  |  |  |  | R14 |
| *Segment 8 (NS)* | | | | | | | |
| ID | **Target sequence** | **H1N1** | **PD09** | **H3N2** | **H5N1** | **H7N9** | **Ref.** |
| – | CGGCTTCGCCGAGATCAGA | *X* |  | *X* | *X* | *X* | R4 |
| – | TGATAACACAGTTCGAGTC | *X* |  | *X* | *X* | *X* | R4 |

Effective *Duals*:

1. S1.2215.19 + S1.2242.15 (5-S and 3-S)
2. S1.2222.15 + S1.2242.15 (5-S and 3-S)
3. S1.2258.15 + S1.2242.15 (3-S)

Effective *Doubles*:

1. S1.2242.15 + S2.2235.19 (5-S and 3-S)
2. S2.2235.19 + S7.52.15 (5-S and 3-S)
3. S1.2215.19 + S7.52.15 (5-S and 3-S)
4. S1.2222.15 + S7.52.15 (5-S and 3-S)
5. S1.2242.15 + S7.52.15 (5-S and 3-S)
6. S1.2237.15 + S7.52.15 (3-S)
7. S1.2252.15 + S7.52.15 (3-S)
8. S1.2258.15 + S7.52.15 (3-S)
9. S1.2252.21 + S7.52.15 (3-S)

Target sequence graphs:

References for Text S3:

1. Ge Q, McManus MT, Nguyen T, Shen CH, Sharp PA, Eisen HN, Chen J. RNA interference of influenza virus production by directly targeting mRNA for degradation and indirectly inhibiting all viral RNA transcription (2003) Proc Natl Acad Sci 100:2718.
2. Sui HY, Zhao GY, Huang JD, Jin DY, Yuen KY, Zheng BJ. Small Interfering RNA Targeting M2 Gene Induces Effective and Long Term Inhibition of Influenza A Virus Replication (2009) PLoS One 4:e5671.
3. Giannecchini S, Clausi V, Nosi D, Azzi A. Oligonucleotides derived from the packaging signal at the 5’ end of the viral PB2 segment speciﬁcally inhibit inﬂuenza virus in vitro (2009) Arch Virol 154:821.
4. Takahashi T, Ohzawa T, Sawada S, Kato N, Goto N, Nakamura S, Yasunaga T, Kaihatsu K. Inhibition of influenza virus infection by targeting genome conserved region with non-natural nucleic acid (2009) Nucleic Acids Symp Ser (Oxf) 53:285.
5. Kwok T, Helfer H, Alam MI, Heinrich J, Pavlovic J, Moelling K. Inhibition of influenza A virus replication by short double-stranded oligodeoxynucleotides (2009) Arch Virol 154:109.
6. Zhang W, Wang CY, Yang ST, Qin C, Hu JL, Xia XZ. Inhibition of highly pathogenic avian influenza virus H5N1 replication by the small interfering RNA targeting polymerase A genes (2009) Biochem Biophys Res Commun 390:421.
7. Abrahamyan A, Nagy E, Golovan SP. Human H1 promoter expressed short hairpin RNAs (shRNAs) suppress avian influenza virus replication in chicken CH-SAH and canine MDCK cells (2009) Antiviral Res 84:159.
8. Zhou K, He H, Wu Y, Duan M. RNA interference of avian influenza virus H5N1 by inhibiting viral mRNA with siRNA expression plasmids (2008) J Biotechnol 135:140.
9. Zhou H, Jin M, Yu Z, Xu X, Peng Y, Wu H, Liu J, Liu H, Cao S, Chen H. Effective small interfering RNAs targeting matrix and nucleocapsid protein gene inhibit influenza A virus replication in cells and mice (2007) Antiviral Res 76:186.
10. Hui EK, Yap EM, An DS, Chen IS, Nayak DP. Inhibition of influenza virus matrix (M1) protein expression and virus replication by U6 promoter-driven and lentivirus-mediated delivery of siRNA (2004) J Gen Virol 85:1877.
11. McCown M, Diamond MS, Pekosz A. The utility of siRNA transcripts produced by RNA polymerase I in down regulating viral gene expression and replication of negative- and positive-strand RNA viruses (2003) Virology 313:514.
12. Zhang T, Wang TC, Zhao PS, Liang M, Gao YW, Yang ST, Qin C, Wang CY, Xia XZ. Antisense oligonucleotides targeting the RNA binding region of the NP gene inhibit replication of highly pathogenic avian influenza virus H5N1 (2011) Int Immunopharmacol 11:2057.
