# Supplementary figures and images for: Discovery of Influenza A Virus Sequence Pairs and Their Combinations for Simultaneous Heterosubtypic Targeting that Hedge against Antiviral Resistance
Source: PLoS Comput Biol. 2016 Jan 15;12(1):e1004663. doi: 10.1371/journal.pcbi.1004663 (PMC4714944; doi:10.1371/journal.pcbi.1004663)

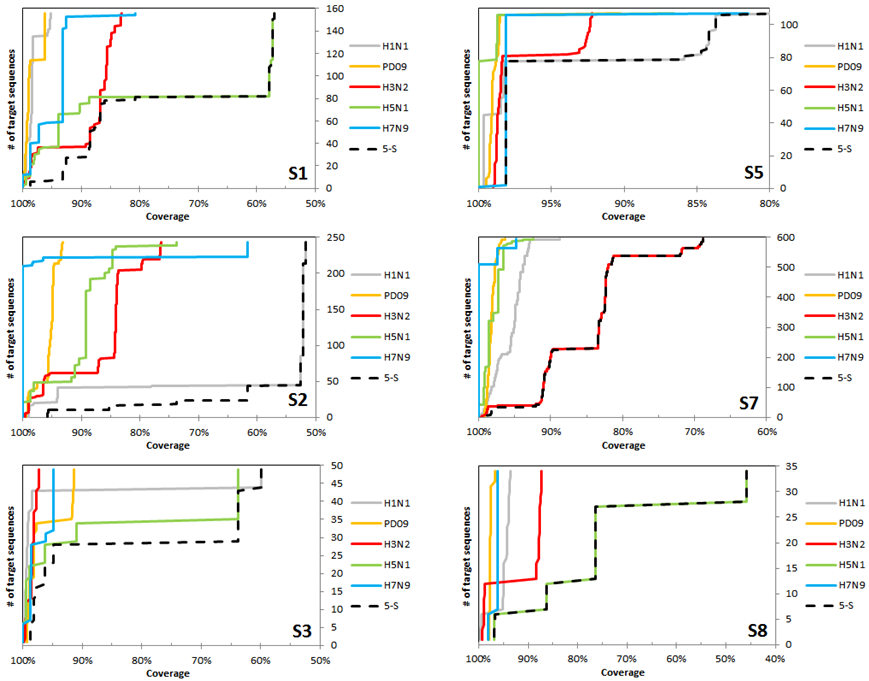

Supplement: S1 Fig — In the graphs, each data point denotes the cumulative number of target sequences (vertical axis) in a particular target segment with a minimum coverage (horizontal axis). The coverage of a target sequence is defined as the percentage of unique segment sequences in the corresponding target segment from subtypes H1N1 (grey), PD09 (orange), H3N2 (red), H5N1 (green), H7N9 (blue) and all the five subtypes (black) in which a match with the target sequence was found. In the occasional incident that the target site of a unique sequence contains an ambiguous base, it is processed by the following rules. The unique sequence is not considered a match when all possible bases of its ambiguous base do not match the respective base of the target sequence; for instance, a K (denotes either G or T) ambiguity code is found at the unique sequence where it is a C at the corresponding target sequence. Otherwise, the unique sequence is omitted during the computation of target sequence coverage when one of the possible bases of its ambiguous base matches the respective base of the target sequence (i.e. the unique sequence is neither a match nor a no-match; for instance, a K (denotes either G or T) ambiguity code is found at the unique sequence where it is a T at the corresponding target sequence. (TIF) [file pcbi.1004663.s010.tif]

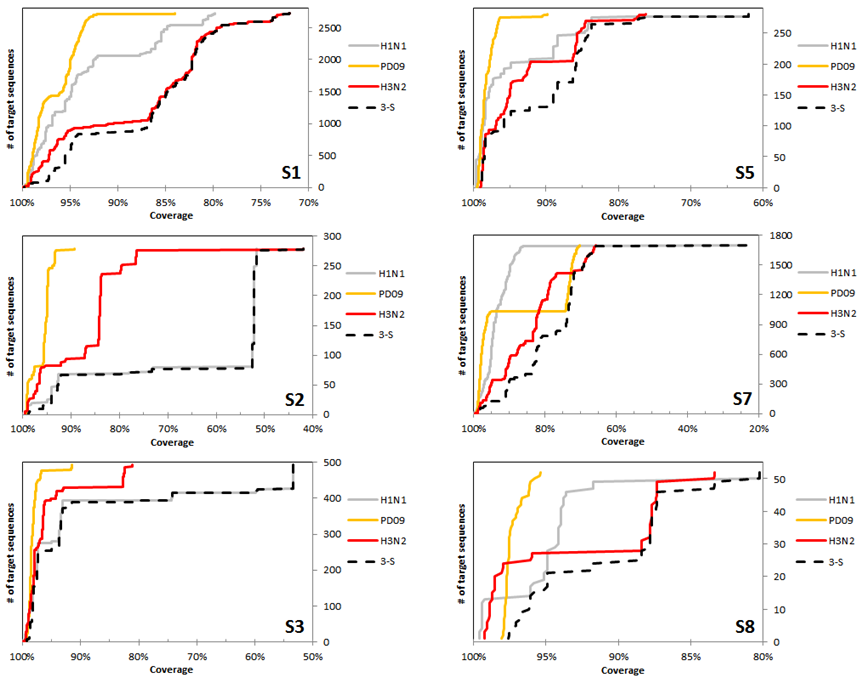

Supplement: S2 Fig — Refer to S1 Fig legend. In this case, only unique sequences from 3 subtypes (H1N1, PD09 and H3N2) were used. (TIF) [file pcbi.1004663.s011.tif]

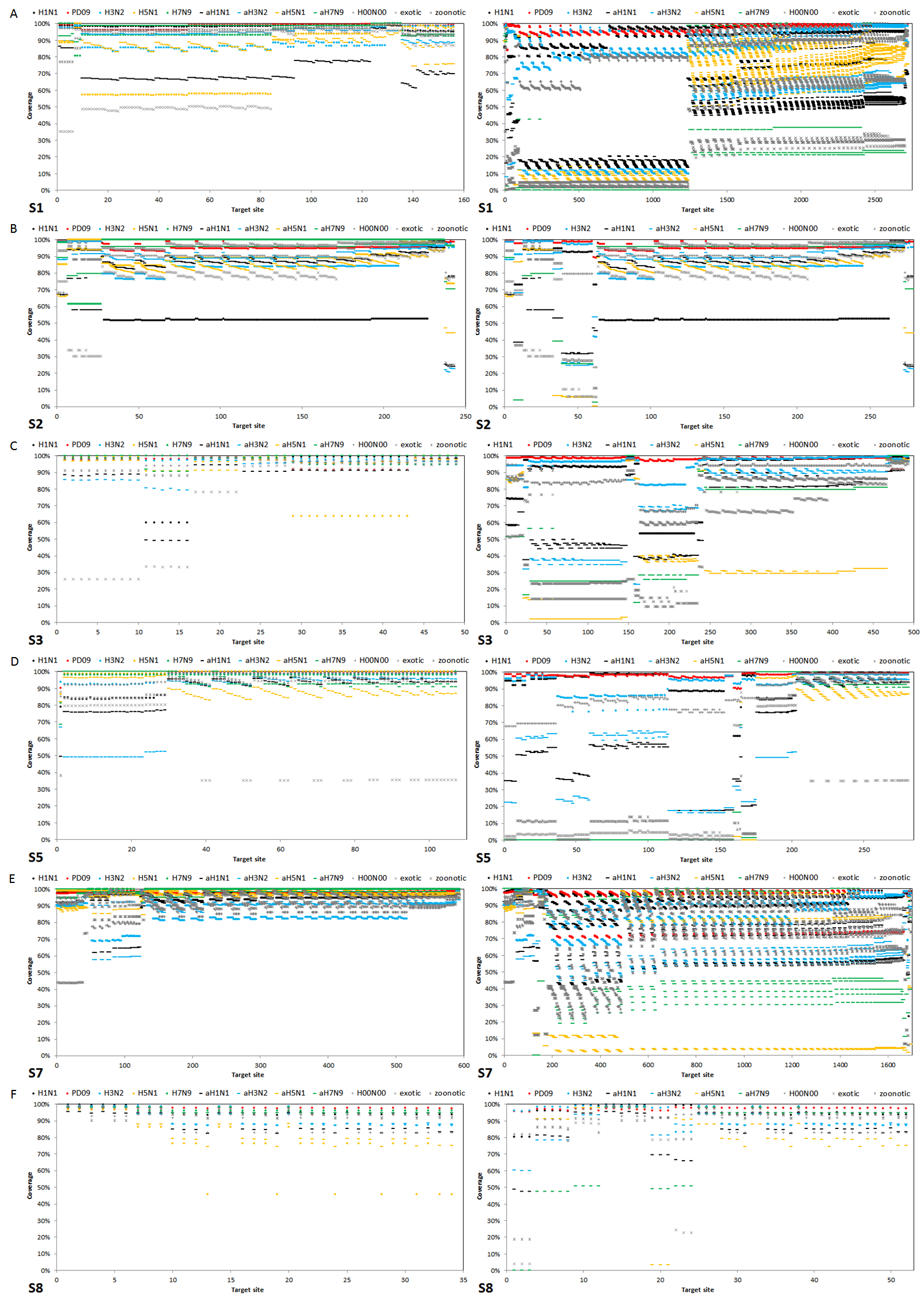

Supplement: S3 Fig — Coverage of each target sequence against subtypes H1N1, PD09, H3N2, H5N1, H7N9, aH1N1, aH3N2, aH5N1 and aH7N9, and against a collection of subtypes grouped as H00N00, zoonotic and exotic (refer to Materials and Methods in the main paper); refer to S1 Fig legend on the procedure to determine the coverage. For plotting purposes (left panel: 5-S; right panel: 3-S), all the target sequences in a segment were numbered (horizontal axis) after they were sorted ascendingly by their coordinates in the target segment followed by their target sequence length. (A) Segment 1. (B) Segment 2. (C) Segment 3. (D) Segment 5. (E) Segment 7. (F) Segment 8. (TIF) [file pcbi.1004663.s012.tif]

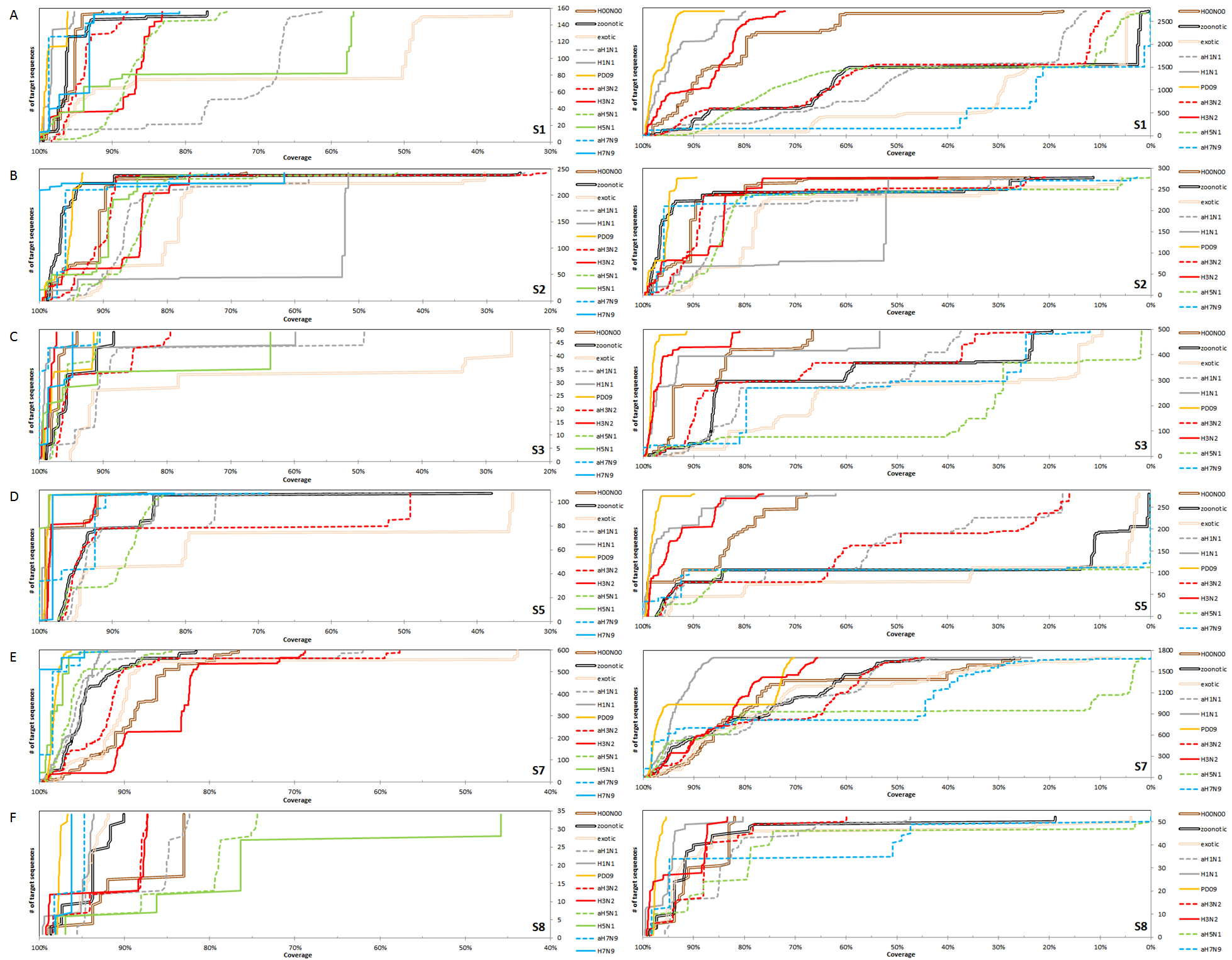

Supplement: S4 Fig — In the graphs (left panel: 5-S; right panel: 3-S), each data point denotes the cumulative number of target sequences (vertical axis) in a particular target segment with a minimum coverage (horizontal axis). Coverage of each target sequence against subtypes H1N1, PD09, H3N2, H5N1, H7N9, aH1N1, aH3N2, aH5N1 and aH7N9, and against a collection of subtypes grouped as H00N00, zoonotic and exotic (refer to Materials and Methods in the main paper); refer to S1 Fig legend on the procedure to determine the coverage. (A) Segment 1. (B) Segment 2. (C) Segment 3. (D) Segment 5. (E) Segment 7. (F) Segment 8. (TIF) [file pcbi.1004663.s013.tif]

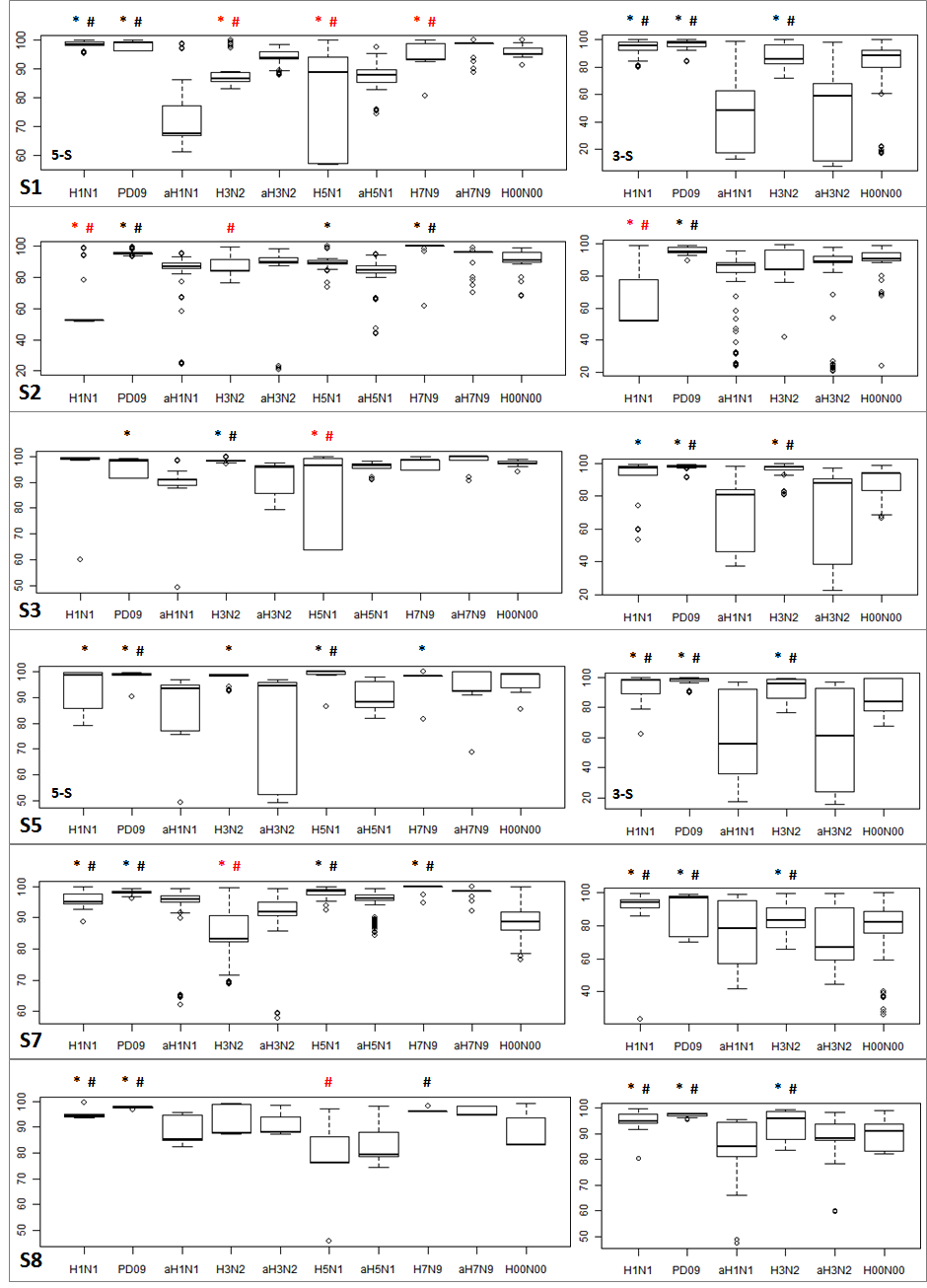

Supplement: S5 Fig — Differences in the coverage distribution of target sequences in each human subtype and in its corresponding animal subtype or in the H00N00 group were tested for statistical significance. Coverage distributions of target sequences in different subtypes were compared by boxplots (vertical axis) and student-t test for 5-S (left) and 3-S (right) sets. One-sided student-t test was performed on the target sequences coverages against each human subtype and against its corresponding animal subtype (i.e. H1N1 vs. aH1N1, PD09 vs. aH1N1, H3N2 vs. aH3N2, H5N1 vs. aH5N1, and H7N9 vs. aH7N9), and against every human subtype and against the H00N00 group of human subtypes (i.e. H1N1 vs. H00N00, PD09 vs. H00N00, H3N2 vs. H00N00, H5N1 vs. H00N00, and H7N9 vs. H00N00). Differences between two coverage distributions were considered as statistically significant when p-value ≤ 0.001. * denotes coverage distribution in the human subtype and the corresponding animal subtype is different (black: coverage distribution in the human subtype is statistically higher; red: coverage distribution in the animal subtype is statistically higher). # denotes coverage distribution in the human subtype and H00N00 group is different (black: coverage distribution in the human subtype is statistically higher; red: coverage distribution in the H00N00 group is statistically higher). Except for segment 5, coverage distribution in the human subtype is not always the highest. Particularly in the 5-S set, more incidences where coverage distribution in the human subtype is either similar to or lower than its corresponding animal subtype or the H00N00 group are observed. (TIF) [file pcbi.1004663.s014.tif]

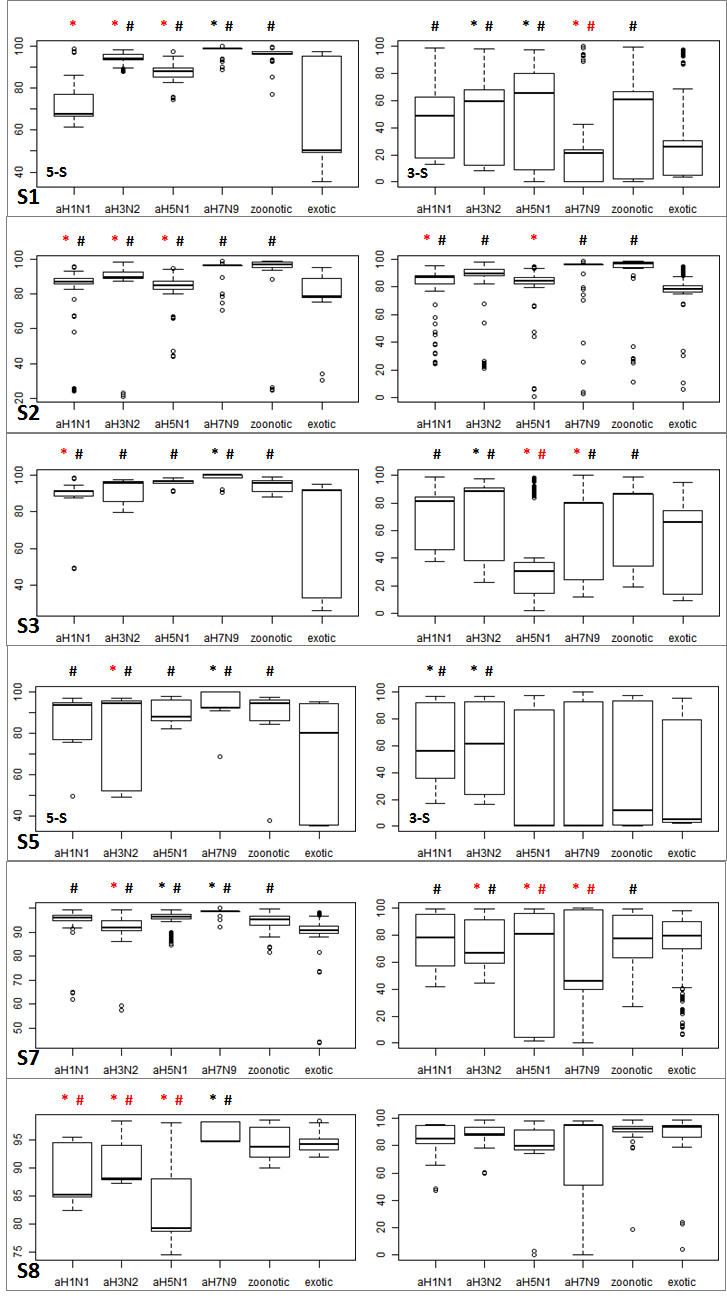

Supplement: S6 Fig — Differences in the coverage distribution in each human corresponding animal subtype and in the zoonotic or exotic groups of animal subtypes were tested. Coverage distributions of target sequences in different subtypes were compared by boxplots (vertical axis) and student-t test for 5-S (left) and 3-S (right) sets. One-sided student-t test was performed on the target sequences coverages against every animal subtype and against the zoonotic group of animal subtypes (i.e. aH1N1 vs. zoonotic, aH3N2 vs. zoonotic, aH5N1 vs. zoonotic, and aH7N9 vs. zoonotic), and against every animal subtype and against the exotic group of animal subtypes (i.e. aH1N1 vs. exotic, aH3N2 vs. exotic, aH5N1 vs. exotic, and aH7N9 vs. exotic), and against the zoonotic and exotic groups. Differences between two coverage distributions were considered as statistically significant for p-value ≤ 0.001. * denotes coverage distribution in the animal subtype and zoonotic group is different (black: coverage distribution in the animal subtype is statistically higher; red: coverage distribution in the zoonotic group is statistically higher). # denotes coverage distribution in the animal subtype or zoonotic group and exotic group is different (black: coverage distribution in the animal subtype or zoonotic group is statistically higher; red: coverage distribution in the exotic group is statistically higher). More incidences where coverage distribution in the human corresponding animal subtype is either similar to or lower than in the zoonotic group are observed for the 5-S set. Coverage distribution in the exotic group is generally the lowest. (TIF) [file pcbi.1004663.s015.tif]

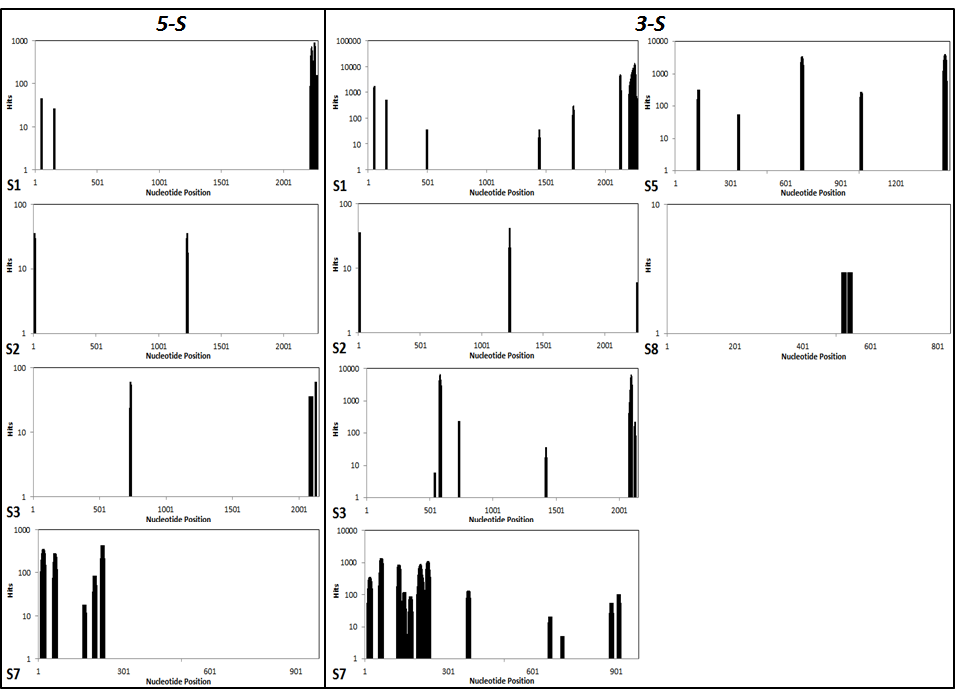

Supplement: S7 Fig — Effective Duals in both 5-S and 3-S sets refer to pairs of single target sequences that can cover all unique sequences of respective target segments. No effective Dual was found for segments 5 and 8 of the 5-S set. (TIF) [file pcbi.1004663.s016.tif]

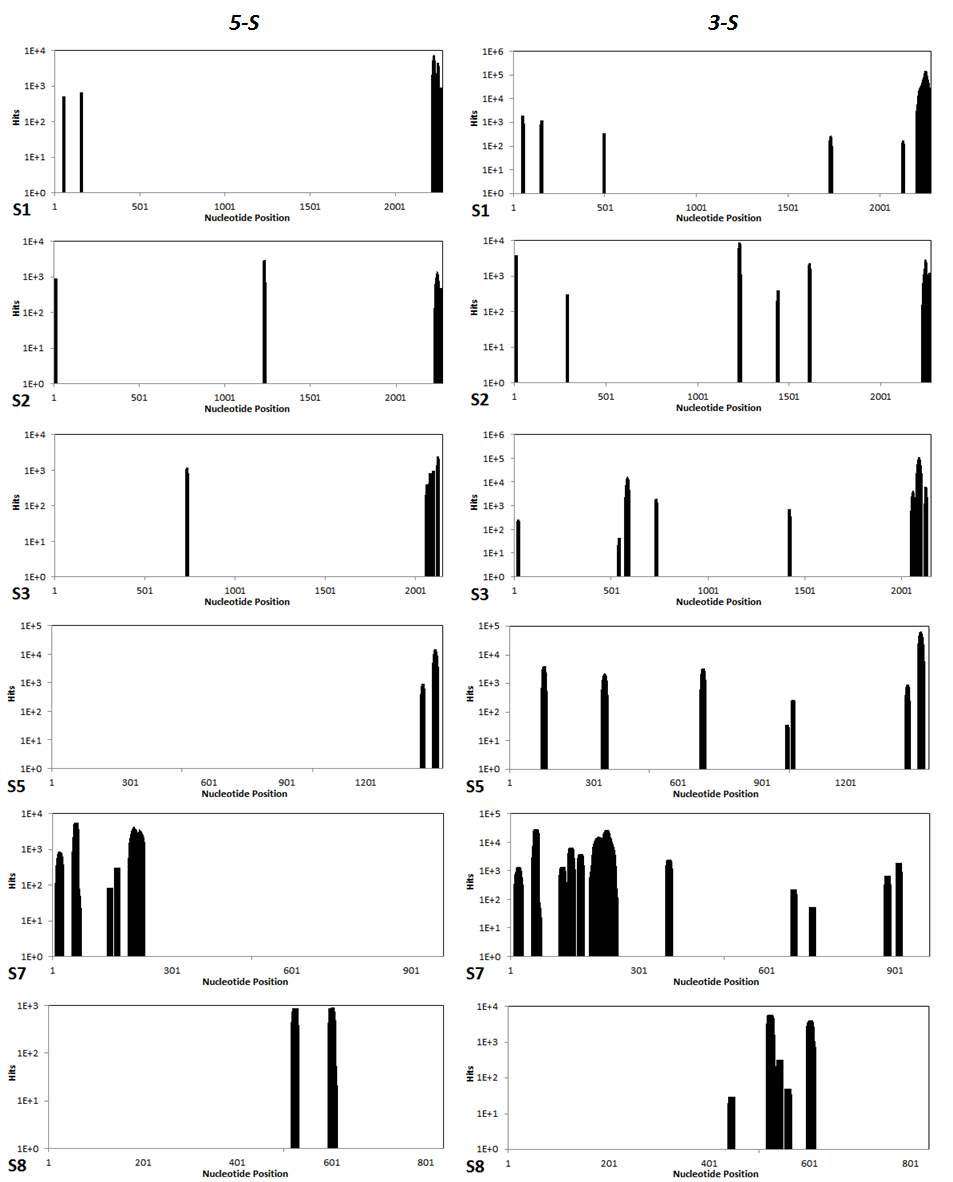

Supplement: S8 Fig — Effective Doubles in both 5-S and 3-S sets refer to pairs of single target sequences in different segment that can cover all virus strains. An effective Double is considered to cover a virus strain when one or both of its target sequences is found in either one or both of the virus strain’s targeted segment sequences. The target sequence position distribution depicted is aggregated from all effective Doubles target sequences obtained from all possible target segment pairings. (TIF) [file pcbi.1004663.s017.tif]

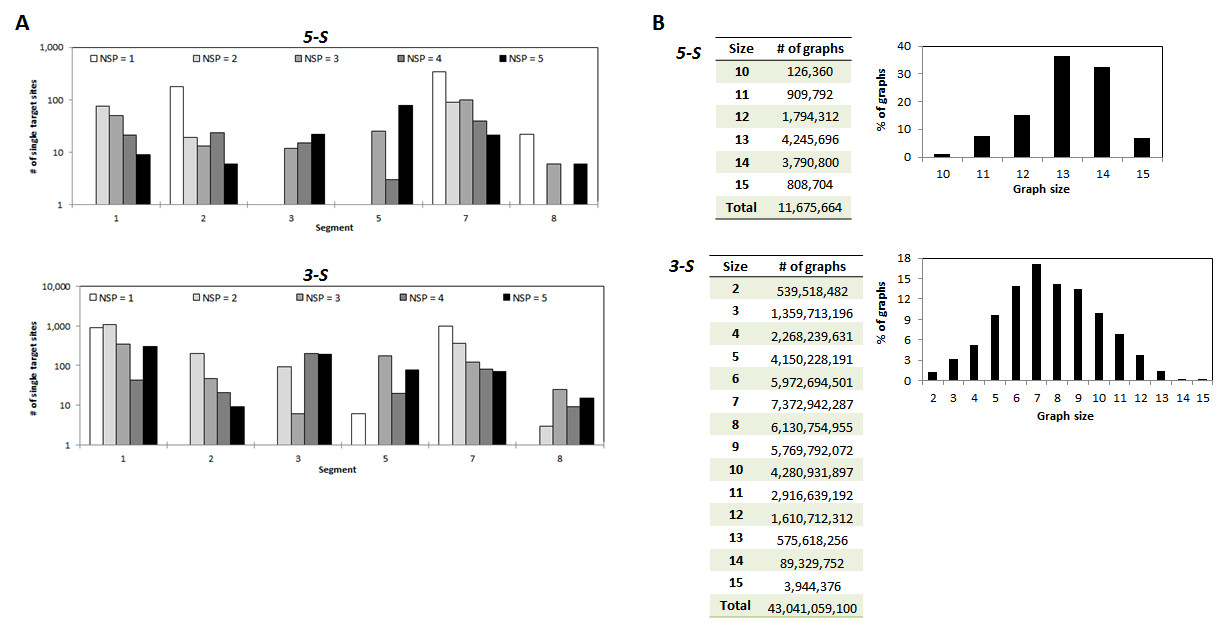

Supplement: S9 Fig — (A) NSP frequency distributions. Number of single target sequences against NSP by target segment in 5-S (top) and 3-S (bottom) sets plotted as bar charts. (B) 6-vertices (NSP = 5) segment partner graphs. The size (number of effective Doubles) distribution of all permutations of 6-vertices segment partner graph constructed by single target sequences with NSP = 5 from the six internal segments were tabulated, and plotted in percentage of total graph permutations for 5-S (top) and 3-S (bottom) sets. (TIF) [file pcbi.1004663.s018.tif]

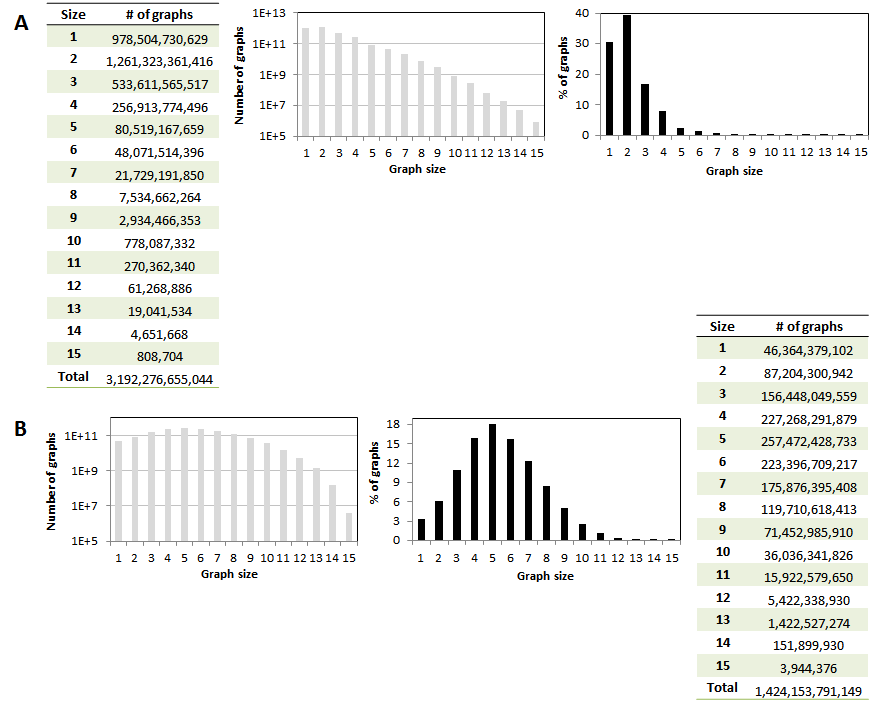

Supplement: S10 Fig — The size (number of effective Doubles) distribution of all permutations of 6-vertices segment partner graph were tabulated (left), and plotted in absolute number of graphs (gray) and in percentage of total graph permutations (black). Graphs were constructed by single target sequences with (A) NSP ≥ 1 (5-S set) and (B) NSP ≥ 4 (3-S set) from the six target segments. Note: determination of graph size becomes computationally intractable in the 3-S set when single target sequences with NSP ≤ 3 are considered, as a consequence of immense total graph permutations. (TIF) [file pcbi.1004663.s019.tif]

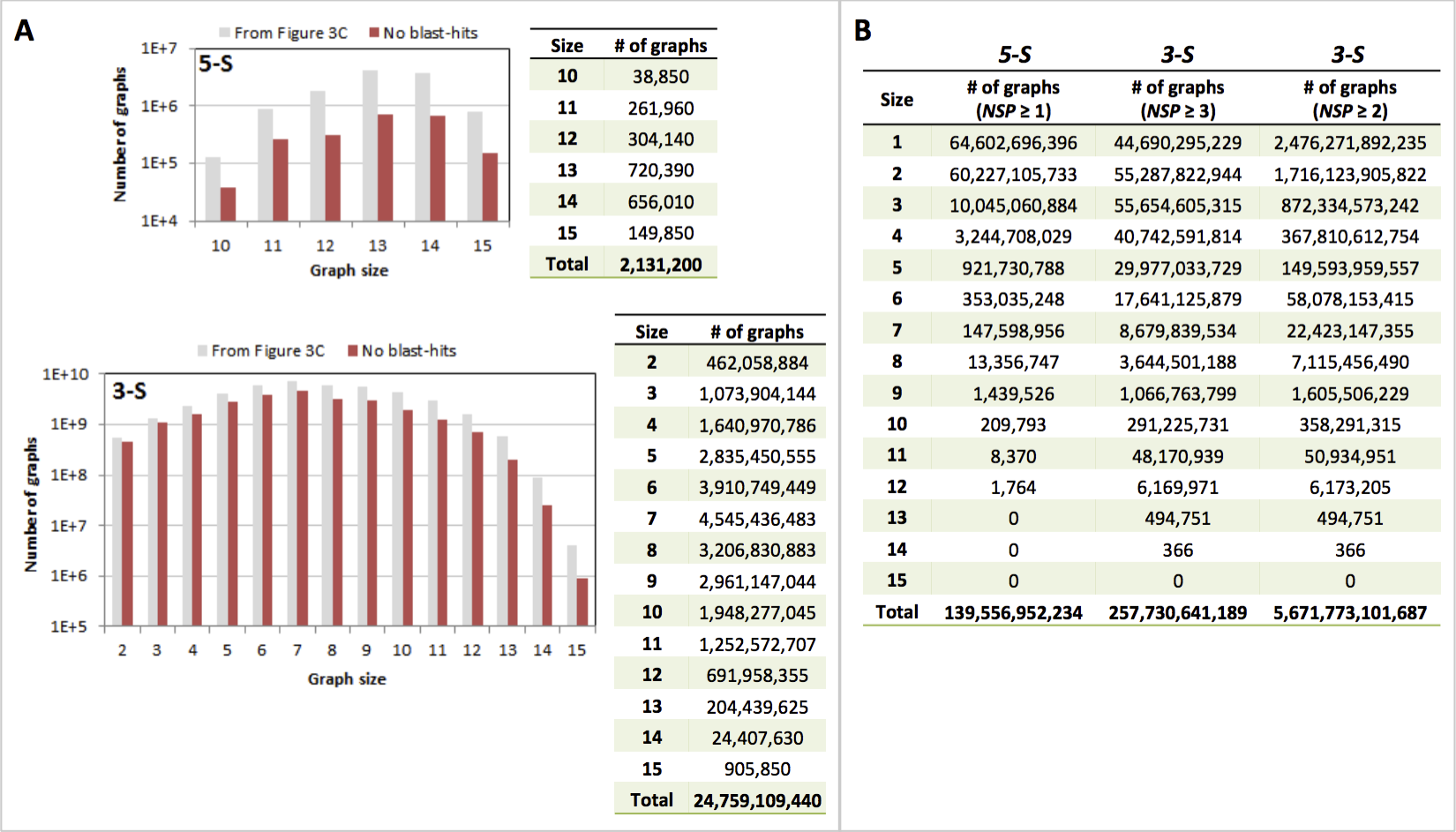

Supplement: S11 Fig — (A) Size distribution of all 6-vertices segment partner graphs formed by a target sequence (whose NSP = 5) from each of the six internal segments (Fig 3C and S9B Fig) in 5-S (top panel) and 3-S (bottom) sets, after the removal of hits with the human transcriptome. The key results remain qualitatively unchanged–the modal number of effective Doubles per graph in 5-S and 3-S is 13 and 7 respectively, every graph in 5-S has at least 10 effective Doubles, and complete graphs (size = 15) that has the highest hedge-factor of five in both sets are still aplenty. (B) Upon removal of target sequences that hit the human genome or transcriptome, the size distribution of all 6-vertices segment partner graphs formed by a target sequence from each of the six internal segments in 5-S (column 1, NSP ≥ 1) and 3-S (columns 2 and 3, NSP ≥ 3 and NSP ≥ 2 respectively) sets. There are respectively 588 and 436,614 complete graphs of 5-vertices formed by a target sequence from S1, S2, S3, S5 and S7 (size = 10) in 5-S and 3-S sets. (TIFF) [file pcbi.1004663.s020.tiff]

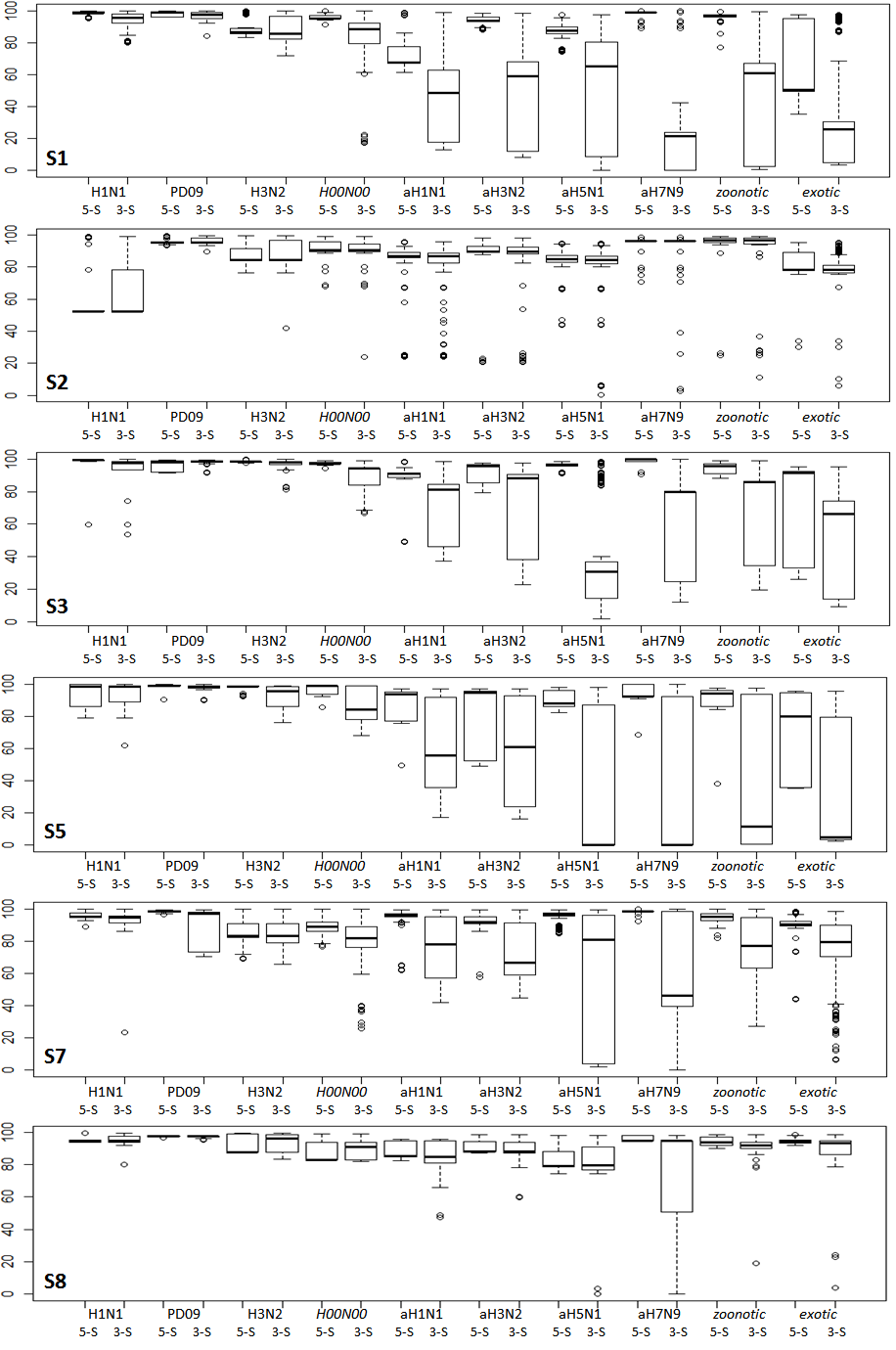

Supplement: S12 Fig — Coverage distributions of target sequences in H1N1, PD09, H3N2, H00N00, aH1N1, aH3N2, aH5N1, aH7N9, zoonotic and exotic were each plotted for 5-S and 3-S sets side-by-side. (TIF) [file pcbi.1004663.s021.tif]

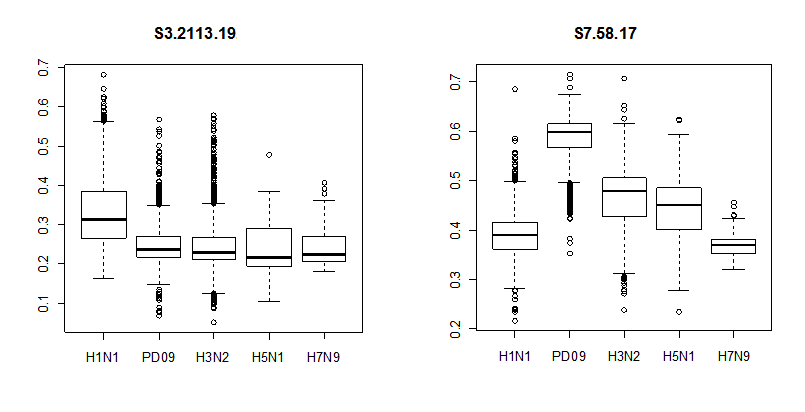

Supplement: S13 Fig — Binding accessibilities of the two representative target sequences in every strain were computed (refer to S1 Text), and their distributions in each of the five human subtypes were depicted as boxplots. Due to variations in the segment sequence among the strains, differences in the segment mRNA co-transcriptional secondary structures can lead to different binding accessibility distributions (right) or have no considerable effect (left). (TIF) [file pcbi.1004663.s022.tif]

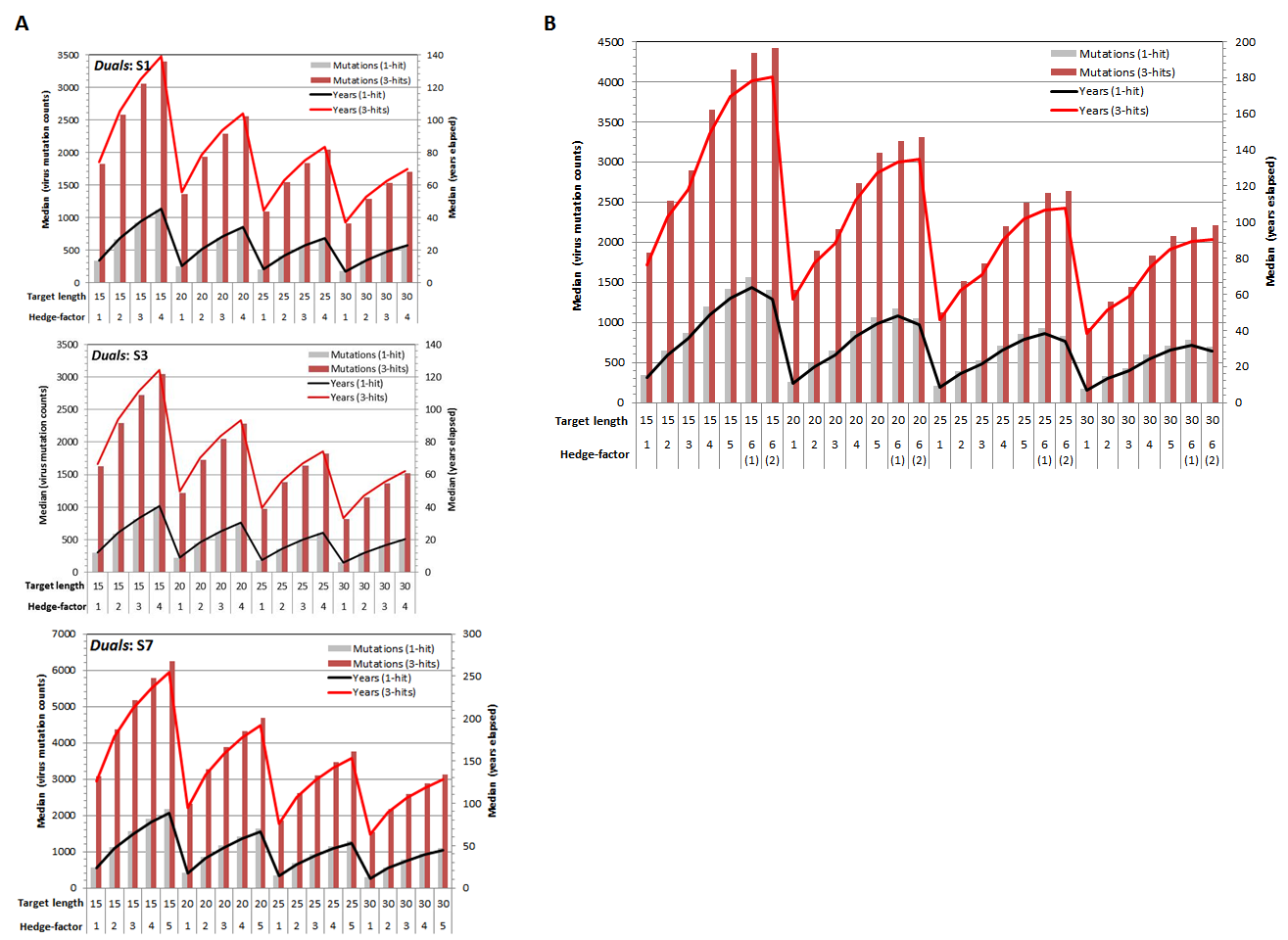

Supplement: S14 Fig — The Monte Carlo simulations described in the main text and shown in Fig 5 were repeated with the condition that a target sequence is considered resistant when it acquires three substitution mutations (3-hits). For ease of comparison with Fig 5, the results labelled as “1-hit” were plotted together with 3-hits. Refer to Fig 5 legend. (TIF) [file pcbi.1004663.s023.tif]
